# Genotyping and lipid profiling of 601 cultivated sunflower lines reveals novel genetic determinants of oil fatty acid content

**DOI:** 10.1186/s12864-021-07768-y

**Published:** 2021-07-05

**Authors:** Alina I. Chernova, Rim F. Gubaev, Anupam Singh, Katrina Sherbina, Svetlana V. Goryunova, Elena U. Martynova, Denis V. Goryunov, Stepan V. Boldyrev, Anna A. Vanyushkina, Nikolay A. Anikanov, Elena A. Stekolshchikova, Ekaterina A. Yushina, Yakov N. Demurin, Zhanna M. Mukhina, Vera A. Gavrilova, Irina N. Anisimova, Yulia I. Karabitsina, Natalia V. Alpatieva, Peter L. Chang, Philipp Khaitovich, Pavel V. Mazin, Sergey V. Nuzhdin

**Affiliations:** 1 Skolkovo Institute of Science and Technology (Skoltech), Bolshoy Boulevard 30, bld. 1, Moscow, 121205 Russia; 2 LLC “OIL GENE”, Skolkovo Innovation Center, Moscow, Russia; 3 Department of Biological Sciences, University of Southern California, Los Angeles, CA 90089 USA; 4 Vavilov Institute of General Genetics, Russian Academy of Sciences, Gubkin st. 3, Moscow, 119991 Russia; 5 FSBSI Lorch Potato Research Institute, Lorkha Str. 23, Kraskovo, 140051 Russia; 6 MSU A.N. Belozersky Institute of Physico-Chemical Biology, Leninsky Gori 1, Building 40, Moscow, 119992 Russia; 7 FSBSI N P Bochkov Research Center of Medical Genetics, Moskvorechye St.1, Moscow, 115522 Russia; 8 Pustovoit All-Russia Research Institute of Oilseed Crops, Filatova St. 17, Krasnodar, 350038 Russia; 9 All-Russia Rice Research Institute, Krasnodar, 350921 Russia; 10 N. I. Vavilov Research Institute of Plant Genetic Resources (VIR), 42 B. Morskaja, St. Petersburg, 190000 Russia

**Keywords:** Sunflower, Genetic markers, UPLC-MS, GBS, GWAS, Fatty acids, Triglycerides

## Abstract

**Background:**

Sunflower is an important oilseed crop domesticated in North America approximately 4000 years ago. During the last century, oil content in sunflower was under strong selection. Further improvement of oil properties achieved by modulating its fatty acid composition is one of the main directions in modern oilseed crop breeding.

**Results:**

We searched for the genetic basis of fatty acid content variation by genotyping 601 inbred sunflower lines and assessing their lipid and fatty acid composition. Our genome-wide association analysis based on the genotypes for 15,483 SNPs and the concentrations of 23 fatty acids, including minor fatty acids, revealed significant genetic associations for eleven of them.

Identified genomic regions included the loci involved in rare fatty acids variation on chromosomes 3 and 14, explaining up to 34.5% of the total variation of docosanoic acid (22:0) in sunflower oil.

**Conclusions:**

This is the first large scale implementation of high-throughput lipidomic profiling to sunflower germplasm characterization. This study contributes to the genetic characterization of Russian sunflower collections, which made a substantial contribution to the development of sunflower as the oilseed crop worldwide, and provides new insights into the genetic control of oil composition that can be implemented in future studies.

**Supplementary Information:**

The online version contains supplementary material available at 10.1186/s12864-021-07768-y.

## Background

Sunflower is an important oilseed crop, domesticated from wild populations in North America approximately 4000 years ago [1] and introduced into Europe in the sixteenth century. Compared to wild sunflower, domesticated lines show significant differences in branching, flowering time, plant height, and various seed traits, including oil content [2]. The cultivation of sunflower as an oilseed crop dates back to the beginning of the nineteenth century. Russian academician V.S. Pustovoit and his colleagues selected sunflower varieties with higher seed oil content, culminating in developing the Peredovik 11 variety in 1958 with the oil content increased to 51% [3]. Russian sunflower varieties with high oil content formed the basis of sunflower breeding worldwide, leading to global sunflower cultivation for oil [4–6].

Today, sunflower is one of the main oilseed crops [7], ranking fourth in global oil production after palm, soybean, and rapeseed [8]. It is cultivated on 26 million hectares, with an average yield of 1.78 metric tons per hectare [9]. In addition to the food industry, sunflower oil is used for polymer synthesis, as a biofuel source and as an emulsifier or lubricant [10]. Nutritional properties and industrial use of sunflower oil depend heavily on the fatty acid residue composition of the main oil lipids, triacylglycerides (TAGs), and some of the minor lipids [11, 12].

Considering the need for varieties with improved oil properties, developing novel varieties with desired oil composition is one of the main directions in oilseed crop breeding [13]. This work requires careful characterization of oil composition using metabolomics and lipidomics techniques, such as ultra-performance liquid chromatography coupled with mass spectrometry (UPLC-MS). For the past few years, there has been a significant increase in the number of studies implementing these techniques in plants. For instance, LC-MS-based techniques were successfully implemented for profiling more than 260 polar metabolites and non-polar leaf lipids in *Arabidopsis thaliana* [14], as well as for the characterization of polar metabolites and lipids in the agronomics plants like tobacco [15], potato [16], corn [17], soybean [18], sunflower [19], and others [20].

Genome-wide association studies (GWAS) coupled with high-throughput lipidome phenotyping can identify genetic variants associated with fatty acid content in sunflower seeds. This knowledge will aid genome-based selection in sunflower breeding programs and speed up the selection of genotypes providing desired fatty acid composition [10]. With rapid development of high-throughput phenotyping and genotyping approaches, as well as the availability of well-assembled and annotated genomes there is a dramatic increase in the understanding of the genetic basis of oil composition in major oilseed crops like soybean and rapeseed [21, 22]. Although sunflower is an important oilseed plant and its oil composition is one of the key agricultural traits, most of the current association studies based on high-throughput genotyping techniques in sunflowere are focused on understanding the genetic control of classical agricultural phenotypes. These studies indeed succeeded in the identification of genetic loci associated with flowering time [23–25], male fertility restoration [26], seedling growth [27], the plasticity of oil yield for combined abiotic stresses [28], basal and apical branching [29], and flower morphology [30] traits. Further, functional analysis of sunflower genome mapped 429 sunflower genes involved in 125 chemical reactions corresponding to 12 oil biosynthesis pathways [31].

Lipidome profiling by UPLC-MS has already been used as an input in GWAS for several plants, for example, maize [32]. However, in sunflower, associated regions have only been identified for some of the most abundant FAs [33–35]. Other minor fatty acids constituting sunflower oil lipids, however, were not considered for association mapping. Genomic predictions have been made for the general oil content trait, but not for the individual components of the oil [36]. The presently documented natural diversity of lipids contained in seeds demonstrates that domesticated oilseed crops like sunflower can serve as a source of rare FAs. This highlights the importance of high-throughput lipid phenotyping, which in combination with the genotype data generates significant potential for oil improvement by customizing its content [37].

In this study, we combined high-resolution lipidome phenotyping and genome-wide genotyping of 601 inbred sunflower lines to perform GWAS to identify genetic variants associated with fatty acid composition-. Furthermore, this work has substantially widened the analyzed sunflower genetic diversity by incorporating the genotypes from Vavilov germplasm collections [38], which nearly doubles the number of inbred lines ever used in sunflower GWAS [30]. Our analysis yielded genetic variants and candidate genes associated with eleven fatty acids, including five minor ones, which have not been previously analyzed.

## Results

### GBS sequencing and SNP calling

We extracted DNA from inbred sunflower lines from the Vavilov seed bank, VNIIMK Applied Agricultural Institute, and Agroplasma Breeding Company collections (Table S1). On average, three technical replicates for each sample were sequenced on the Illumina HiSeq 4000 platform using a GBS protocol (see *Experimental Procedures*), resulting in 1490 genotypes (601 unique genotypes, some of them were resequenced once, some twice). Reads were mapped onto the *Helianthus annuus* reference genome (HanXRQr1.0), with the mapping rates varying between 75 and 90%. Variant calling identified 2,360,111 single nucleotide polymorphisms (SNPs) spanning all 17 chromosomes. Homozygosity and Principal Component Analysis (PCA) showed no obvious bias due to plate, batch or seed bank variables (Figure S1A-B). Pairwise comparisons showed that technical replicates grouped together, and samples have low discordance with other technical replicates (Figure S1C).

### Population structure, relative kinship, and linkage disequilibrium

**Fig. 1:**
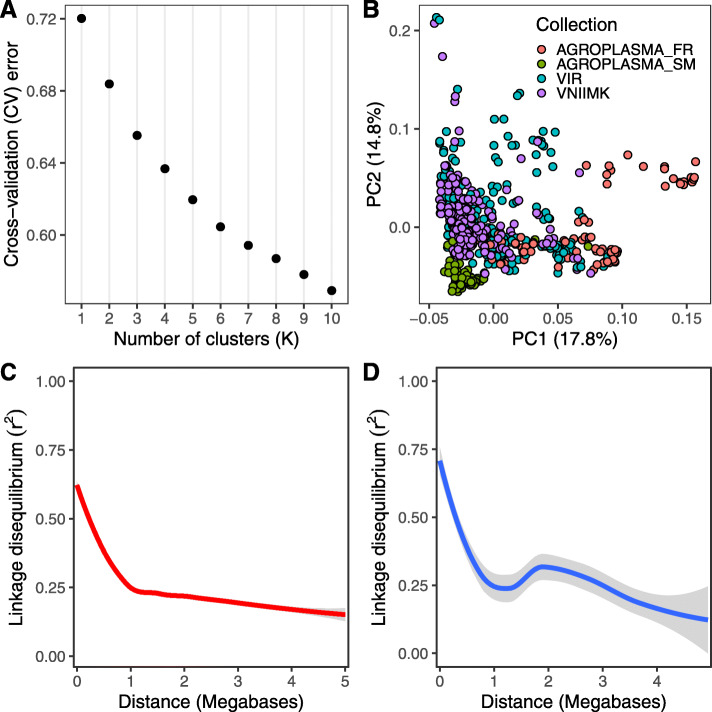
Population structure of germplasm and linkage disequilibrium (LD) values. **A** Estimated cross-validation error value for possible cluster number from 1 to 10. **B** Subpopulations were assessed using Principal Component Analysis. Each dot corresponds to a sunflower accession used in the study. Color corresponds to sunflower lines from different collections. Agroplasma_SM indicates sterility maintainer lines from Agroplasma; Agroplasma_FR indicates fertility restorer lines. **C**, **D** Genome-wide (**C**) and per-chromosome 3 (**D**) LD-decay. Lines correspond to loess curves

We assessed the population structure of the genotypes used in the GWAS analysis using the ADMIXTURE package. No visible clusters were observed in the cases of K = 1:10 (Fig. 1A). However, visualization of genetic variation using the first two principal components of PCA revealed a distinct group of genotypes derived from the Agroplasma collection (See Samples in Experimental Procedures) which clustered separately (Fig. 1B). While the average genotype correlation (r^2^) dropped to half of its maximum value at 0.7 Mb, linkage disequilibrium (LD) decay varied among the 17 chromosomes (Fig. 1C, D; Figure S2A-B). Notably, some chromosomes, such as chromosome 3, demonstrated extended LD within the 1–3 Mb interval (ANOVA, *p* < 0.0001).

### Genotypes variability and relation to other sunflower germplasms

**Fig. 2:**
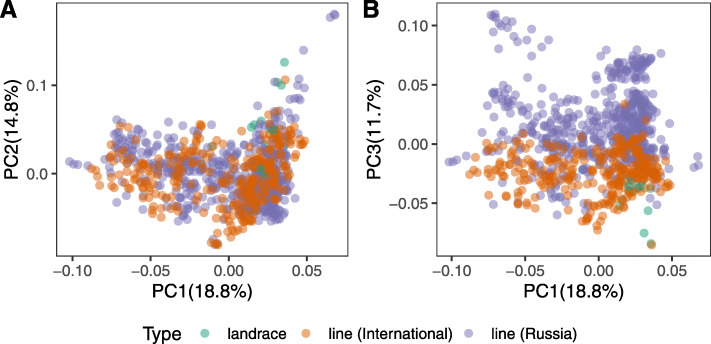
The relationship between sunflower germplasm of different origins estimated based on 2345 SNPs shared between this and the Hübner (2019) studies. **A** The first and the second PCA components. **B** The first and the third PCA components. Each dot corresponds to a plant accession. Colors indicate the origin

To place the analyzed cultivars on a broader map of sunflower genotype variation, we compared our genotypes to the previously sequenced 1065 wild sunflower varieties, 20 landraces, and 289 cultivated sunflower lines [39]. Principal component analysis using just cultivated lines and landraces based on 2345 SNPs shared between the datasets showed that cultivated sunflower lines from the Russian dataset are distinguishable from those collected worldwide by the third principal component (Fig. 2A, B). The analysis further reaffirmed the broad genetic difference between cultivated and wild material (Figure S3A). However, it has to be noted that this analysis is confined to the genomic positions polymorphic in both datasets and could, therefore, underestimate the differences between them. The third principle component further separated some of the *Helianthus* species (Figure S3B). The landraces present in the Hübner dataset mostly clustered between the cultivated lines from both foreign and Russian collections, and the wild sunflower varieties (Figure S3A, B).

### Oil lipidome quantification

We extracted the total lipid fraction from the sunflower seeds of the same 601 sunflower lines used in the genotype analysis. We then divided the lipid extracts into two fractions and analyzed them independently using UPLC-MS technology. The first fraction was kept intact, while the second was hydrolyzed before the analysis. The hydrolyzed fraction contained fatty acid residues of all oil lipids and a minor fraction of free fatty acids present in the intact samples before hydrolysis (FAs). Mass-spectrometry analysis yielded 826 computationally annotated lipid peaks and 27 post-hydrolysis fatty acids. We focused on a specific lipid class, the triacylglycerides (TAGs), which is the most important among sunflower oil lipids. To optimize the detection of both low and high abundance FAs and TAGs, we conducted the UPLC-MS measurements at two extract dilutions (see *Experimental Procedures*).

### Quantification of genetic and environmental effects on oil lipidome composition

**Fig. 3:**
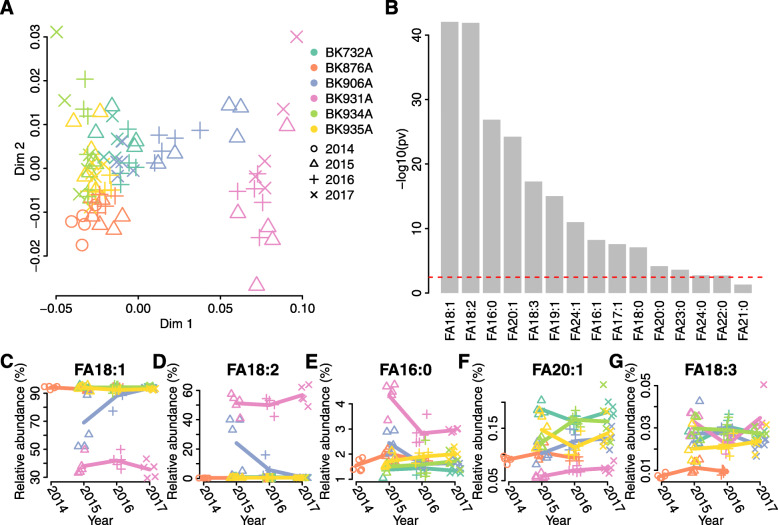
FAs concentrations in replication experiments. **A** M ultidimensional scaling plot (two dimensions, 1 - Spearman correlation coefficient between FAs abundances was used as distance). One sample is shown by one point; accessions are shown by different colors; different years are shown by points of different shapes. **B** Minus log10 *p*-values for the differences between lines (ANOVA) are shown, FAs are ordered by p-value increase. Bonferroni adjusted 0.05 significance level is shown by red line; **C**-**G**) abundances of oleic acid (18:1) (**C**), linoleic acid (18:2) (**D**),) palmitic acid (16:0) (**E**), eicosenoic acid (20:1) (**F**), and linolenic acid (18:3) (**G**) are shown across lines and years. Each point represents 1 sample, point shapes, and colors as in (**A**), lines show per-year averages

To assess the environmental and biological reproducibility of FA and TAG data, we grew plants of six sunflower inbreed lines (1 conventional and 5 high oleic) originating from the VNIIMK collection. The genotypes were grown for 3 years with five biological replicates per year and yielded a total of 89 data points (Table S1). We performed genotyping using the same GBS protocol and collected the UPLC-MS measurements at different extract dilutions to ensure quantitative coverage of the entire concentration range. We then tested the effects of the genotype by environment interaction (GXE) using ANOVA with the following model: G + E + GXE (where G is genotype and E is environment, i.e. year). All FAs and TAGs measured in both dilutions displayed significant differences between genotypes after BH-correction (*p* < 0.05, Figs. 3А, S4А, S5A, S6, S7, S8, Table S2). FAs (11 out of 15) and TAGs (32 out of 42 and 43 out of 59 for 1:25 and 1:3 dilutions, respectively) also showed significant GXE interaction. The interaction effect, although statistically significant, had a much smaller amplitude than the effect of the genotype (Figure S9). Biological replicates of the same genotype collected in different years displayed greater similarity than those collected in the same year (Figs. 3А, S4А, S5A). The strongest variation among genotypes was observed for oleic, linoleic and palmitic acids, the major fatty acids in sunflower oil (Fig. 3B-G), as well as for the following TAGs: 50:2, 51:3, 54:3, 54:4, 54:6 (Figure S4 B-G and S5 B-G). Analysis of Nei’s genetic distances between genotypes obtained for the same seeds used for lipidomic analysis showed the reproducibility of genotypes between biological replicates (Table S3).

### Oil lipidome variation analysis

**Fig. 4:**
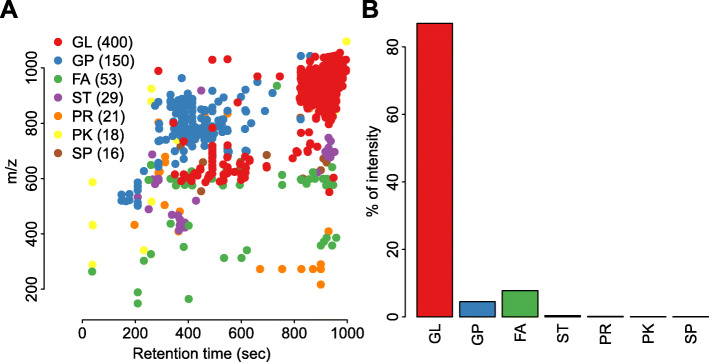
Lipid annotation. **A** Mz/rt. plot. One point represents one peak; different lipid categories are shown in different colors. Only peaks with sample intensities at least two times higher than blank intensities are shown. **B** Relative intensities of all lipid categories. The intensity of a given category was calculated as the sum of intensities of all lipids in the category. GL- glycerolipids, GP- glycophospholipids, FA -fatty acids, ST- sterols, PR- prenols, PK- polyketides and SP-saccharolipids

**Fig. 5:**
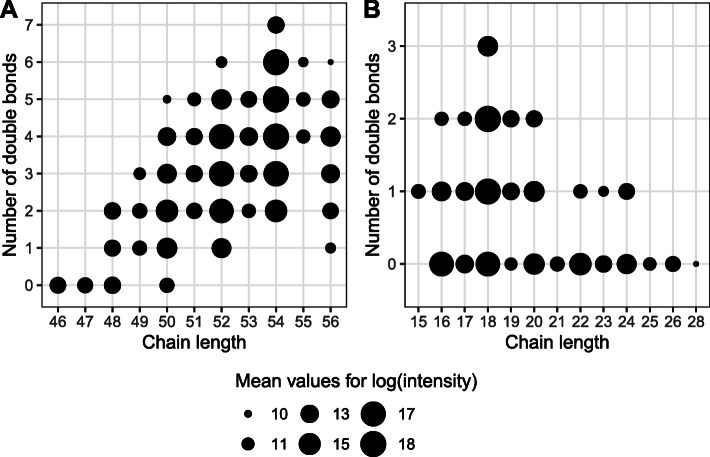
Schematic representation of fatty acid properties (fatty acid chain length and degree of saturation) for detected lipids. **A** Cumulative chain length and double bonds number of the three fatty acid residues composing the detected TAG molecules. **B** Chain length and double bonds number of fatty acid (FAs) released after lipid hydrolysis. Each circle corresponds to a FA or a TAG. The circles’ size corresponds to the mean relative amount of this molecule in a sample (log-transformed MS peak intensity)

Computational annotation of the intact lipidome of the oil samples extracted from 601 sunflower lines yielded 687 lipids falling into seven lipid classes: glycerolipids (GL), glycophospholipids (GP), free fatty acids (FA), sterols (ST), prenols (PR), polyketides (PK), and saccharolipids (SP) (Fig. 4A). A subclass of glycerolipids, TAGs, constituted 87% of lipid intensities of uniquely identified compounds (Fig. 4B, Table S4). The most abundant TAGs were 54:6, 54:5, 54:4, 54:3, 52:4, 52:3, and 52:2 (Fig. 5A). Among computationally annotated 27 FAs, the most enriched FAs were 18:1, 18:2, 16:0, and 18:0 (Fig. 5B). The statistics on each of the fatty acid are presented in Table S5.

### Association analysis

**Fig. 6:**
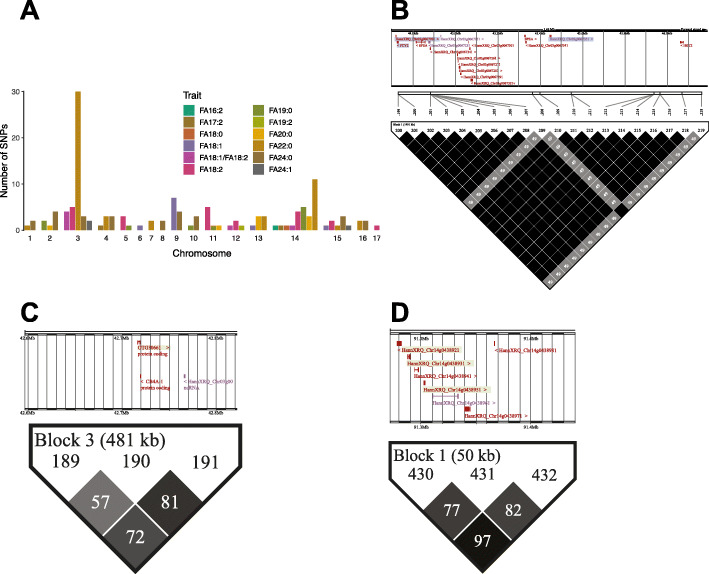
GWAS results for FAs in sunflower lines and candidate genes for docosanoic acid improvement. **A** Cumulative plot representing the number of significant associations for each of the traits. Traits represented by colors. Chromosome number and the number of SNPs are presented on the X and Y-axes, respectively. **B** LD block in Chr3 (Location 44,696,624–46,188,263). **C** LD block in Chr3 (Location 42,596,595–43,078,214). **D** LD block in Chr14 (Location 91,496,885–91,547,710). Candidate genes in blue associated with lipid metabolism, Candidate genes in green associated with lipid metabolism described by Badouin et.al (2017) [31]

Of the 601 sunflower lines taken into the study, both genotype and lipid intensity data for a total of 543 accessions was obtained. We conducted GWAS analysis using the mixed linear model (MLM) approach to test for genetic determinants of FAs variation based on this data. A total of 15,068 SNPs - passed the filtering criteria (missing calls rate < 0.3, DP > 4, MAF > 0.01) for oleic and linoleic acids and 12,528 SNPs for other fatty acids (missing calls rate < 0.3, DP > 4, MAF > 0.03). From 27 detected FAs, 23 satisfying the criteria for GWAS were selected. We detected significant associations for eleven FAs: stearic acid (18:0), oleic acid (18:1), linoleic acid (18:2), nonadecanoic acid (19:0), eicosanoic acid (20:0), docosanoic acid (22:0), tetracosanoic acid (24:0), tetracosenoic acid (24:1), and hexadecadienoic acid (16:2) and rare fatty acids such as 17:2 and 19:2 (MLM, Bonferroni-corrected *p* < 0.00001; Fig. 6; Figure S10A-F). We further performed GWAS for the oleic-linoleic acid ratio yielding six significant SNPs - (Bonferroni-corrected *p* < 0.00001; Figure S10G-I). Altogether, we identified 140 trait-associated SNPs (MLM, Bonferroni-corrected *p* < 0. 0.00001; Fig. 6A). Among them, docosanoic acid (22:0) - showed the strongest association with 53 SNPs located on chromosomes 3 and 14 (Table S6) explaining - 35.4% of the quantitative variation of docosanoic acid abundance in sunflower lines.

### SNP annotation and candidate gene identification

To annotate genes potentially linked to the genetic variants significantly associated with FAs quantitative variation, we determined the boundaries of the corresponding LD blocks (Figure S11, Table 1). We then retrieved the annotations of all genes located within these LD blocks and checked their intersection with the genes annotated to be involved in sunflower oil metabolism [31] (Table S7). Of the 44,144 genes annotated in sunflower – 429 genes have been reported to be involved in oil metabolism [31]. - 124 of these genes are located close to significant SNPs and four of them coincide with the genes reported in Badouin’s list [31], which is significantly more than expected by chance (Fisher exact test, *p* = 0.03, odds ratio = 3.4). According to the sunflower genome annotation, these genes encode putative beta-hydroxyacyl-(acyl-carrier-protein) dehydratase FabZ, HotDog domain protein, probable phosphatidic acid phosphatase (PAP2) family protein, and putative MYB-CC type transcription factor, LHEQLE-containing domain and are located on Chr3 and Chr14 (Fig. 6C, D).

**Table 1:** LD blocks with significant associations

Phenotype	Chromosome	LD block Location		Length (kb)
		Start position	End position	
Oleic Acid (18:1)	6	64,066,219	64,889,534	823
	9	168,736,699	169,306,761	570
	13	116,940,760	117,370,881	430
	15	38,043,597	38,078,709	35
Linoleic Acid (18:2)	3	66,733,584	68,666,170	932
	5	37,199,838	37,569,381	396
	11	5,004,818	50,619,247	414
	11	95,051,157	92,468,132	416
Linolenic acid (18:3)	11	43,846,946	44,328,722	481
Oleic/Linoleic ratio	3	66,733,584	68,666,170	932
	12	121,534,492	121,906,701	372
Nonadecanoic acid (19:0)	2	179,620,148	179,872,251	252
	14	53,394,600	53,480,813	86
	14	59,829,070	60,503,626	664
Docosanoic acid (22:0)	3	32,332,262	32,562,669	230
	3	42,596,595	43,078,214	481
	3	44,696,624	46,188,263	1491
	3	48,304,030	49,705,352	1401
	3	53,949,047	54,230,339	281
	3	57,635,146	57,714,809	79
	14	91,496,885	91,547,710	50
	14	96,632,645	97,927,934	614
	16	176,846,705	176,869,659	22
Tetracosanoic acid (24:0)	2	56,777,868	56,880,436	102
	2	73,398,255	74,229,960	831
	3	102,040,303	102,070,280	29
Nervonic acid (24:1)	3	44,696,624	46,188,263	1491
	3	57,635,146	57,714,809	79

## Discussion

Our results broaden the list of candidate genes and genetic variants associated with fatty acid composition in sunflower. Our analysis, based on UPLC-MS mass-spectrometry included the quantitative measurements of fatty acids present in sunflower oil in minor amounts, which have not been previously assessed. Our results indicate that there are genetic loci with - substantial effect on - some of these minor fatty acids, such as docosanoic acid (22:0). Observed variation in docosanoic acid concentration between wild-type sunflower accessions paves way into breeding sunflower lines with elevated levels of minor fatty acids in the future [40].

The reason for the lack of significant signals for 12 of 23 analyzed fatty acids as well as relatively weak genetic signals for many of the eleven fatty acids with identified associations could -be due to the chosen sunflower lines. The 543 lines used in the GWAS analysis as well as all the 601 lines used in our study were not preselected to have contrasting phenotypes for fatty acid content, with the exception for oleic and linoleic acids, because until now, only these fatty acids together with stearic and palmitic acids were considered in breeding programs [41]. Another reason for the lack of associations can lie just in the absence of variation within the Helianthus genus for certain FA phenotypes [40]. Nonetheless, our approach involving a large number of diverse cultivated lines yielded enough variability for nine other sunflower fatty acids to produce significant genetic associations. Further work involving lines specifically selected to vary in - fatty acid content is required to determine the full scope of genetic associations underlying sunflower oil composition.

We have identified six large LD blocks containing SNPs significantly associated with FA content - in chromosome 3 (Table 1). Furthermore, among the reported candidate genes predicted to affect oil quality, three genes associated with lipid metabolism localized within the large 1491 kb LD block of chromosome 3 (Figure S11, Table 1). These genes encode the putative phospholipase A2 (this protein releases FAs from the phospholipid), putative CRAL-TRIO lipid-binding domain-containing protein, and putative ethanolamine-phosphate cytidylyltransferase. Predicted functions of these genes, although not yet assessed experimentally in sunflower, single out this genomic region as one of the key regulators of sunflower oil FA composition (Fig. 6B, Table S7). - Among the genes located within the chromosome 14 region associated with FA 22:0 variation, two were annotated as membrane-bound proteins: putative membrane-bound transcription factor site-2 protease, and putative membrane-bound O-acyl transferase (MBOAT). This finding agrees with the fact that very-long-chain fatty acids in sunflower are synthesized by membrane-bound enzymes [42].

Among the genes important for fatty acid metabolism according to Badouin et al. [31] and located within the LD blocks linked to FA variation, one of the most interesting is the gene encoding a putative FabZ dehydratase, the protein responsible for FA elongation (Fig. 6C). Since the genomic resolution of our study is limited to LD blocks, which typically include multiple genes, further work is needed to map associations to specific genes and causative genetic variants.

Genetic variants (SNPs) linked to the oleic-linoleic acids ratio also map to a chromosome 3 region (302 kb region; Figures S10I, S11). This LD block overlaps with the one carrying SNPs significantly associated with linoleic acid content (Figures S10H, S11). This finding supports the notion that genomic regions underlying linoleic acid content should also be involved in oleic-linoleic acids ratio determination. Unfortunately, there were no annotated genes in this region that were known to be directly related to the biosynthesis or modification of fatty acids-. Previous studies demonstrated that genes encoding desaturases, the major enzymes responsible for the oleic-linoleic acids ratio, are located on chromosomes 1, 14 [34]. Nonetheless, loci potentially associated with oleic and linoleic acid contents were previously identified on chromosome 3 by means of QTL mapping [33, 34], as well as by computational predictions [31]. These loci did not overlap with the locus obtained in the current study. There could be a number of reasons why the previously reported regions potentially related to oleic-linoleic acids ratio were not identified in the present study. First, the SNP coverage for these regions might not have been dense enough in our data. Second, previously identified associations might play lesser role in determining linoleic and oleic-linoleic acid ratio under Russia’s environmental conditions. Third, the lack of the overlap could be related to the specific genetic features of the studied cohort that was restricted to the lines from the Russian collections.

In addition to the genetic variants linked to oleic-linoleic acids ratio, we have identified 9 and 22 SNPs significantly associated with individual oleic and linoleic acid content, respectively. These SNPs localized on chromosomes 9, 13, 15 for oleic acid and 3, 11, 12, 14, 15, and 17 for linoleic acid. Previously, a study reported QTLs identified using ORS markers for oleic acid content on chromosomes 8 and 9, and linoleic acid content on chromosomes 8 and 14 [43]. In addition, previous studies reported a QTL for oil and phytosterol content on chromosome 14 [44]. We have also identified significant associations and putative candidate genes on these chromosomes for linoleic acid and on chromosome 9 for oleic acid. However, our chromosome 9 LD block did not overlap with the QTL associated with oleate reported by Badouin et al. [31].

Interestingly, we have additionally identified a putative FAO1 gene on chromosome 9 as a candidate for oleic acid abundance. This is a long-chain fatty alcohol oxidase gene involved in the omega-oxidation pathway of lipid degradation [45]. For minor FAs, we identified a large LD block on chromosome 14 containing the associations with docosanoic and noncosanoic acids, in line with the computational predictions of Badoun et al. [31]. All the genes which were previously described in association with sunflower lipid metabolism and SNPs in these genes could be good candidates for further functional studies.

## Conclusion

This is the first large-scale study on Russian sunflower germplasm, which will make a significant contribution to sunflower development as the oilseed crop worldwide. Comparison of the Russian sunflower lines with the data on the cultivated and wild sunflower published by Hübner et al. 2019 [39] showed that Russian sunflower germplasm contains unique variation which is not presented in the international collections.

As a result of climate change, sunflower can become the leading plant in oil production because of its ability to grow under different environmental conditions [46]. In this view, sunflower varieties with oil properties customized for specific applications may become in-demand in the future. Our study makes a step in this direction by identifying genetic associations for both major and minor FAs present in sunflower oil. Genetic markers for minor FAs, such as docosanoic and noncosanoic acids, have not been previously studied. We hope that future sunflower breeding programs will benefit from understanding the genetics governing the ratios of these oil components important for industrial applications.

## Methods

### Samples

Sunflower samples were provided by N.I. Vavilov Institute of Plant Genetic Resources (VIR, St. Petersburg, Russia) (https://www.elibrary.ru/item.asp?id=32542976; http://www.vir.nw.ru/en/), V.S. Pustovoit All-Russia Research Institute of Oilseed Crops (VNIIMK)(https://vniimk.ru/science/geneticheskaya-kollektsiya-podsolnechnika/), and LLC Agroplasma Seed and Breeding Company (Krasnodar, Russia) (https://agroplazma.com/contacts). Samples are accessible upon request. (https://agroplazma.com/contacts).

Two hundred ninety-two (255 were sequenced) inbred lines from N.I. Vavilov Institute of Plant Genetic Resources (VIR, St. Petersburg, Russia) are mostly conventional lines in terms of fatty acid composition (18:2 range from 36 to 79%), 3 middle – oleic (18:1 > 50%), 1 high-oleic line (18:1 > 80%).

One hundred ninety-nine inbred lines were from V.S. Pustovoit All-Russian Research Institute of Oilseed Crops (VNIIMK) (Krasnodar, Russia). Fatty acid composition is known for 99 lines: 2 lines high-oleic (18:1 > 80%), 7 middle – oleic (18:1 > 50%), other lines with 18:2 range between 36 and 70%.

One hundred forty-seven oil-producing sunflower lines were provided by Agroplasma Seed and Breeding Company (Krasnodar, Russia). Fatty acid composition is unknown.

All plants were grown and seeds collected in the Krasnodar region in Russia. For UPLC-MS analysis seeds themselves were used. For DNA extraction, seeds were germinated in the lab. All the reagents can be found in Methods S1.

All lines are diploid, 2n = 34. All seeds were stored for 1–3 years. All lines were obtained by at least 8 rounds of self-pollination.

Plants were grown in field in the middle part of the Krasnodar Region.

Soils of the leached black earth soil type. Sunflower was sowed following the preceding crop, fall wheat, at the seeding rate of 40,000 plants per hectare.

Sowing was carried out according to the following sowing system: 70 × 35 cm, a single plant per planting pit. Farming techniques, as commonly used for sunflower.

Each line was grown on the plot with an area of 9.1 m^2.^

Details on the dataset can be found in Additional file 23: Appendix S1. More information can be found in VIR and VNIIMK databases, Agroplasma is a private company, but information can be achieved by a special request. Also, information on VIR genealogy was previously published [38, 47].

Plants for environmental studies experiment (6 lines) were selected from VNIIMK dataset. They were grown on the regular basis by collection holders at one location: The Central Krasnodar Region, GPS coordinates 45°04′50′’ N and 39°04′57.′

### DNA extraction

DNA was extracted from chlorophyll-free sprouts after 1 week of germination without light. 100 mg of tissue for each sample was grounded to powder using FastPrep-96™ Automated Homogenizer (MP Biomedicals). Total DNA was extracted according to the CTAB protocol using the NucleoSpin® Plant II plant DNA extraction kit (Macherey-Nagel, Germany) and stored at − 20 °C until needed. The purified DNA sample quality and concentration were determined by gel electrophoresis and Qubit 3.0 Fluorometer (Thermo Fisher Scientific, USA).

### GBS library preparation

Illumina libraries were constructed using two restriction endonucleases – HindIII (rarely cutting enzyme, A/AGCTT) and NlaIII (frequently cutting, CATG/) according to the protocol described by [48] with minor modifications. Details of the method are provided in Methods S2.

### GBS sequencing and primary data analysis

Each 96-multiplexed library was sequenced across three lanes in Illumina HiSeq 4000 (San Diego, CA, USA) at the Skoltech Genomics Core Facility as either 150 bp or 75 bp paired-end reads. The sequencing dataset can be found in NCBI repository: https://www.ncbi.nlm.nih.gov/bioproject/620114 [49]. Illumina reads were mapped onto the *Helianthus annuus* reference genome HanXRQr1.0 [31] using BWA MEM 0.7.9a-r786 [50] with consideration for uniquely mapped reads whose PE ends mapped within 1 K of each other. Variants were called using the GATK pipeline, which considers indel realignment and base quality score recalibration and calls variants across all samples simultaneously through the HaplotypeCaller implemented in GATK. Variants were filtered using hard filtering parameters: MQ > 36, QD > 24, and MQRankSum< 2, ensuring that reads were mapped to a unique place in the reference with high quality (MQ), that the reads carrying both alleles were comparable in terms of mapping quality (MQRankSum), and that the actual variants were called with high quality (QD), filters that were not applied by default by GATK’s HaplotypeCaller, resulting in the 2.3 M SNP calls. To retain SNPs for population and GWAS analyses for oleic and linoleic acids missing calls rate < 0.3, DP > 4, MAF > 0.01 were applied, resulting in 15,068 SNPs, for GWAS for other fatty acids more strict MAF > 0.03 was used resulting in 12,528 SNPs.

VCF file with SNP variants is provided in the supporting information (missing calls rate < 0.1, DP > 4, MAF > 0.01).

Validation of SNP calls was performed using the MALDI-TOF MS technology (Agena Bioscience’s MALDI-TOF-based scalable MassARRAY). 75 lines were selected and re-genotyped for 11 SNPs, which were significantly associated with at least one studied trait and with MAF higher than 0.05 in the 75 selected lines (Table S8). For six SNPs, genotypes identified by the two technologies were completely identical across all the lines studied. For the remaining five lines, the proportion of lines with identical genotypes varied from 0.91 to 0.95. For eight SNPs, the agreement between two methods was statistically significant (Permutation test, BH-corrected *p* < 0.05).

### Population structure

Genetic diversity of analyzed lines was estimated using PCA with the aid of PLINK (http://pngu.mgh.harvard.edu/purcell/plink/) [51] based on 15,068 SNPs with minor allele frequency (MAF) > 0.01 called on all 17 chromosomes. Population structure was analyzed using ADMIXTURE v1.3.0 [52] with the number of clusters varying from 1 to 10. To compare the dataset from Hübner et al. 2019 [39] with the lines analyzed in the current study, common SNPs between 2 datasets were identified by merging 2 datasets. The merging procedures were performed using the bcftools’s functions isec and merge. The merged SNPs were filtered using the MAF filter of 0.03.

### Linkage disequilibrium

LD was estimated across the sunflower genome using VCFtools [53] to calculate frequency correlation (r^2^) between 25,431 biallelic SNPs with MAF > 0.03 whose genotypes were supported by at least 4 reads called in at least 60% of individuals.

### Lipid extraction

For lipid extraction, 10 mg (for each line) of sunflower seeds (1 sample-1 seed) with 400 uL of methanol/methyl tert-butyl ether mixture (1:3 v:v) were homogenized in Precellys evolution (Bertin corp. USA) (6800 rpm, 3* 20 s, pause 30 s) coupled with Cryolis filled with dry ice with 6 2.8 mm zirconium oxide beads (Bertin corp. USA) at the temperature not higher than 10 degrees. Then, extraction was performed using methanol/methyl tert-butyl ether mixture, according to [54] with minor modifications (Methods S3).

For FAs analysis, the extracts obtained in the previous steps were hydrolyzed using the protocol adopted from [55] (Methods S3).

### UPLC-MS profiling

Samples were processed using mass-spectrometry coupled with reversed-phase ultra-performance liquid chromatography (UPLC-MS) (ACQUITY UPLC System; Waters, USA) in positive and negative ionization modes in Q-TOF Maxis Impact II, Bruker Daltonik, Germany. Settings: Ion Polarity: positive/negative, Scan mode: MS, Mass range: 50 -1200 m/z, Spectra rate: 2 Hz.

UPLC separation was performed on the C8 Acquity Beh column (2.1 mm Х 100 mm, 1.7-μm particle size; Waters) and the Acquity BEH C8 1.7 μm Vanguard precolumn (Waters) at 60 °C.

The detailed information can be found in Methods S3.

Previously we have validated the extraction and profiling technique for FAs [56] and TAGs [57] in sunflower.

### Lipidomic data analysis and annotation

For data processing, optimal parameters were generated using the Bioconductor IPO package. The subsequent peak peaking, chromatogram alignment, chemical noise subtraction and intensity thresholding were performed using the XCMS 3.1 package (https://bioconductor.riken.jp/packages/3.1/bioc/html/xcms.html) [58]. The output was a list of peaks, with retention time, m/z, and intensity for each sample. To exclude possible contaminants, mean intensities of all sunflower peaks were compared to mean intensities in blank samples. (Figure S12). Only lipids with sample intensity at least two times higher than blank intensities were used in the analysis.

To annotate FAs and TAGs, formulas of the possible lipids (irrespective to isomers) in these classes were generated. For FAs, chain lengths from С10:0 to С28:0 with not more than 6 double bonds were considered. For TAGs, the total chain length varied between 30 and 85 carbon atoms, and the number of double bonds varied from 0 to 12. Then, the masses of generated lipids were compared to the m/z of detected peaks. For FAs just one adduct (−H^+^) was considered; for TAGs four adducts (H^+^, Na^+^, K^+^, and NH_4_^+^) were considered. All peaks with ppm (ppm = abs(m1 − m2)/max(m1, m2) × 10^6^), where m1 and m2 are the mass of the lipid and m/z of the peak, respectively) below 10 were considered as the possible lipids of the given class. Then, for each of the two lipid classes and for each adduct, peaks were manually filtered based on the assumption that correct FAs and TAGs should form a net-like pattern on the RT-m/z scatter plot. To annotate non-TAG lipids measured in the positive mode, lipid Befdatabase (The LIPID MAPS® Lipidomics Gateway, https://www.lipidmaps.org/) was used. First, all isomers were collapsed, then m/z of all non-TAG peaks were compared with the masses of all lipids from the lipidmap. Same adducts as were used for TAGs were considered. All lipid-peak pairs with ppm < 10 were considered to be a valid annotation. All peaks annotated with lipids of just one category were assigned to this category; peaks annotated with lipids of more than one category were considered as ambiguously annotated.

Reproducibility experiments with 3 years of replicates and the main dataset were measured and processed separately.

In the reproducibility experiment, only TAGs with the NH_4_^+^ adduct and FAs were considered. For both dilutions in the case of TAGs and for FAs, the intensity of individual lipids was divided by the total intensity of all TAGs/FAs in the given sample and multiplied by 100. To assess the role of genetic and environmental factors ANOVA with the following model was used:

$$ \mathrm{Lipid}\_\mathrm{concentration}\sim \mathrm{line}+\mathrm{year}+\mathrm{line}:\mathrm{year}. $$

MDS analysis in two dimensions was performed based on one minus Spearman correlation coefficient distance.

### Association analysis and annotation

Since sunflower genotypes were obtained from three different seed banks, it was important to account for their genetic relatedness and hence the mixed linear model was implemented (MLM: Y = SNP + PCs + Kinship + e, where Y – phenotype, SNP and PCs – fixed effects, Kinship – random effect, e - error). In addition, internal standards intensity and LS-MS batch numbers were used as co-factors to account for the batch effect and sample weight in the model.

Before GWAS, FAs distribution between the samples were estimated (Figure S13). For GWAS, all the samples with 10% and more missing data (for phenotypes) were excluded from the analysis. GWAS was performed using TASSEL 5 [59]. SNPs for the analysis were filtered out using the following criteria: missing calls rate < 0.3, DP < 4, and minor allele frequency (MAF) < 0.01 for such traits as oleic and linoleic acids and MAF < 0.03 for the other traits. Filtering was performed using VCFtools. A mixed linear model was used where the SNP effect and population structure estimated by PCA were treated as fixed effects and kinship was included in the model as a random effect. The genetic relatedness analysis was performed with the relative kinship coefficients (K-matrix) being calculated using the TASSEL software (Centered IBSmethod). The collection and the batch number were also used as factors and sample weight and internal standards intensity as covariates. To estimate the mixed linear model performance, quantile-quantile plots (q-q plots) were used. Observed *p*-values were plotted against the expected probability of their distribution. To represent GWAS results, Manhattan plots were used where p-values were plotted for all sunflower linkage groups one by one. GWAS results were visualized with the help of the qqman R package (version 0.1.4).

To determine the significance of observed hits, 0.05/5000 p-value threshold was used. This is a Bonferroni correction based on the average number of LD blocks. The total number of SNPs used in GWAS was divided by 5000 - the number of LD blocks estimated from LD analysis. LD block analysis was performed using the Haploview software [60]. Gene annotation within each LD block was performed using the sunflower genome browser (https://sunflowergenome.org).

To estimate the variance in docosanoic acid concentration explained by identified 53 SNPs, the linear model with same covariates as was used in GWAS analysis and with all 53 SNPs was used. The results show that these SNPs could explain 35.4% of the variance.

## Supplementary Information

**Additional file 1: FigS1.** ((A) PCA plots reflecting the relationships between sunflower technical samples based on 15,068 SNPs segregating in the Russian collection. Each dot corresponds to a sunflower technical sample used in the study. Dots are colored by sequencing batch (A) or collection samples were obtained from (B) (C) Nei’s genetic distance matrix between each 2 samples.

**Additional file 2: FigureS2.** Linkage disequilibrium (LD) decay plot. (A) Genome-wide LD. Gray dots correspond to a SNP pair, y-axis show r^2^ between two SNPs calculated using whole dataset. SNP pairs with distance less than 5 Mb are shown. (B) LD per each chromosome. Lines correspond to loess curves; 95% confidence intervals are shown by shades.

**Additional file 3: FigureS3.** Joint principal component analysis of sunflower accessions genotyped in this study and in Hübner (2019) based on 2345 shared SNPs. The first and the second (A) or the first and the third (B) PCs are shown. Each dot corresponds to a plant accession. Colors indicate the origin (wild/line/landrace). Shapes indicate species.

**Additional file 4 **(A) Multidimensional scaling plot (two dimensions, 1 - Spearman correlation coefficient between TAGs abundances measured using LC-MS with dilution 1:3 was used as distance). One sample is shown by one point; accessions are shown by different colors; different years are shown by points of different shapes. (B) Minus log10 *p*-values for the differences between lines (ANOVA) are shown, TAGs are ordered by p-value increase from top left to bottom right. Bonferroni adjusted 0.05 significance level is shown by red line; (C-G) abundances of five selected TAGs are shown across lines and years. Each point represents 1 sample, point shapes, and colors as in (A), lines show per-year averages. This figure complements main Fig. 3.

**Additional file 5: FigureS5.** (A) Multidimensional scaling plot (two dimensions, 1 - Spearman correlation coefficient between TAGs abundances measured using LC-MS with dilution 1:25 was used as distance). One sample is shown by one point; accessions are shown by different colors; different years are shown by points of different shapes. (B) Minus log10 p-values for the differences between lines (ANOVA) are shown, TAGs are ordered by p-value increase from top left to bottom right. Bonferroni adjusted 0.05 significance level is shown by red line; (C-G) abundances of five selected TAGs are shown across lines and years. Each point represents 1 sample, point shapes, and colors as in (A), lines show per-year averages. This figure complements main Fig. 3.

**Additional file 6: FigureS6.** Replication experiment on 6 accessions: relative abundances of all the fatty acids with significant (FDR < 0.05) effect of genotype, FAs are ordered by p-value increase. Each point represents 1 sample, point shapes, and colors denote years and accessions, respectively, lines show per-year averages.

**Additional file 7: FigureS7.** Replication experiment on 6 genotypes: relative abundances of all TAGs detected by LC-MS with dilution 1:3 with significant (FDR < 0.05) effect of genotype, TAGs are ordered by p-value increase. Each point represents 1 sample, point shapes, and colors denote years and accessions, respectively, lines show per-year averages.

**Additional file 8: FigureS8.** Replication experiment on 6 genotypes: relative abundances of all TAGs detected by LC-MS with dilution 1:25 with significant (FDR < 0.05) effect of genotype, TAGs are ordered by p-value increase. Each point represents 1 sample, point shapes, and colors denote years and accessions, respectively, lines show per-year averages.

**Additional file 9: FigureS9.** Replication experiment on 6 accessions. (Left) Distributions of FAs (top) or TAGs (middle and bottom) by percentages of variance explained by genotype (G), environment (year, E), interaction between genotype and environment (G:E) or percentage of residual variance. Distribution of the same percentages divided by number of degrees of freedom of corresponding factor are shown on the right.

**Additional file 10: FigureS10.** GWAS Manhattan plots for (a) Stearic acid; (b) Nonadecanoic acid; (c) Eicosenoic acid; (d) Docosanoic acid; (e) Tetracosanoic acid; (f) Nervonic acid; (g) Oleic acid; (h) Linoleic acid; (i) Ratio between oleic and linoleic acids. FDR and Bonferroni thresholds are shown by blue and red, respectively.

**Additional file 11: FigureS11.** LD blocks containing significant SNPs. Each panel represents one significant association, traits and chromosome names where significant loci is located are shown in panel titles. Each panel schematically shows associated loci with all detected SNPs. LD-blocks detected by Haploview software are shown by heatmaps, r^2^ (%) for each SNP pair is shown by color and numbers.

**Additional file 12: FigureS12.** Data clean up using blank samples. Top panels show dependence of average log2 seed sample intensity of FAs and TAGs (two dilutions) on average log2 intensity of same lipids in blank samples (see Methods). Straight and dashed lines correspond to equal intensities in both types of sample and to two-fold higher concentration in seed samples compared to blanks, respectively. Bottom panels show the same lipids as top panels in coordinates of total FA (TAG) chain length (x-axis), and number of double bounds (y-axis), point size is proportional to log2 average intensity in seed samples. Only lipids with log2(sample/blank) > 1 were used in analysis, remaining FAs and TAGs (shown in red) were filtered out.

**Additional file 13: FigureS13.** Distributions of natural logarithm of row LC-MS fatty acids intensities across accessions.

**Additional file 14: TableS1.** The list of samples used in the study.

**Additional file 15: TableS2.** ANOVA *p*-values for FA content in replication experiment.

**Additional file 16: TableS3.** Nei’s genetic distances between samples from replication experiment.

**Additional file 17: TableS4.** Annotation of all LC-MS peaks detected in positive mode. For all peaks lipidmap category is given, “unk” denotes that no relevant annotation was found, “ambig” means that peak has ambiguous annotation on category level. For TAGs annotated more precisely using net-like patterns exact formula, adduct and ppm are provided.

**Additional file 18: TableS5.** Summary statistics on FAs abundances.

**Additional file 19: TableS6.** List of found significant associations.

**Additional file 20: TableS7.** Functional annotation of genes from LD blocks harboring SNPs with significant association with at least one lipid trait.

**Additional file 21: TableS8.** SNP validation using MALDI-TOF. Table provides genotypes determined using MALDI-TOF for selected SNPs in sunset of lines.

**Additional file 22:** Supporting experimental procedures. **Method S1.** Reagents, **Method S2.** GBS library preparation, **Method S3.** Lipid extraction and UPLC-MS.

**Additional file 23: AppendixS1.** Description of the lines used in the study.

**Additional file 24.** maxmiss.7.maf0.01.vcf VCF file with SNP calls.

## Data Availability

The authors confirm that the data supporting this study’s findings are available within the article, its supplementary materials, and public available sources. Accessions catalog names are provided in supplementary information (Table S1) the accessions can be obtained upon request from VIR seed bank (https://www.elibrary.ru/item.asp?id=32542976; http://www.vir.nw.ru/en/), VNIIMK collection (https://vniimk.ru/science/geneticheskaya-kollektsiya-podsolnechnika/) and LLC Agroplasma (https://agroplazma.com/contacts). *Helianthus annuus* reference genome HanXRQr1.0 used in the study is available in NCBI repository: https://www.ncbi.nlm.nih.gov/assembly/GCF_002127325.1/. Sequencing dataset can be found in NCBI repository: https://www.ncbi.nlm.nih.gov/bioproject/620114.

## Abbreviations

GBS: Genotyping-by-sequencing
TAG: Triacylglyceride
FA: Fatty acid
UPLC–MS: Ultra-performance liquid chromatography coupled with Mass-Spectrometry
GWAS: Genome-wide association study
QTL: Quantitative trait locus
VIR: N.I.Vavilov Research Institute of Plant Industry
VNIIMK: Pustovoit All-Russia Research Institute of Oil Crops
PCA: Principal component analysis
LPA: Lysophosphatidic acid
PC: Phosphatidylcholine
PE: Phosphatidylethanolamine
PI: Phosphatidylinositol
PA: Phosphatidic acid
LD: Linkage disequilibrium
MLM: Mixed linear model
MAF: Minor allele frequency

## References

[1] Crites GD (1993). Domesticated sunflower in fifth millennium B.P. temporal context: new evidence from middle Tennessee. Am Antiq.

[2] Burke JM, Tang S, Knapp SJ, Rieseberg LH (2002). Genetic analysis of sunflower domestication. Genetics..

[3] Martínez Force E (2015). Sunflower: chemistry, production, processing, and utilization.

[4] Friedt W (1992). Present state and future prospects of biotechnology in sunflower breeding. Field Crops Res.

[5] Seiler GJ, Rieseberg LH. Systematics, origin, and germplasm resources of the wild and domesticated sunflower. In: Schneiter, AA (ed.) Sunflower Technology and Production, Agronomy Series 35. Madison: American Society of Agronomy Inc; pp. 21–65.

[6] Terzić S, Boniface M-C, Marek L, Alvarez D, Baumann K, Gavrilova V, Joita-Pacureanu M, Sujatha M, Valkova D, Velasco L, Hulke BS, Jocić S, Langlade N, Muños S, Rieseberg L, Seiler G, Vear F (2020). Gene banks for wild and cultivated sunflower genetic resources. OCL..

[7] Dimitrijevic A, Horn R. Sunflower hybrid breeding: from markers to genomic selection. Front Plant Sci. 2018;8. 10.3389/fpls.2017.02238. 10.3389/fpls.2017.02238 PMC5776114 29387071

[8] Rauf S, Jamil N, Tariq SA, Khan M, Kausar M, Kaya Y (2017). Progress in modification of sunflower oil to expand its industrial value. J Sci Food Agric.

[9] Konyalı S (2017). Sunflower production and agricultural policies in Turkey. Sos Bilim Araşt Derg.

[10] Dimitrijević A, Imerovski I, Miladinović D, Cvejić S, Jocić S, Zeremski T, Sakač Z (2017). Oleic acid variation and marker-assisted detection of Pervenets mutation in high- and low-oleic sunflower cross. Crop Breed Appl Biotechnol.

[11] Velasco L, Ruiz-Méndez MV. Sunflower oil minor constituents. In: Sunflower: Elsevier; 2015. p. 297–329. 10.1016/B978-1-893997-94-3.50017-9.

[12] Venegas-Calerón M, Troncoso-Ponce MA, Martínez-Force E. Sunflower oil and lipids biosynthesis. In: Sunflower: Elsevier; 2015. p. 259–95. 10.1016/B978-1-893997-94-3.50016-7.

[13] Jocic’ S, Miladinovic’ D, Kaya Y. Breeding and Genetics of Sunflower. In: Sunflower: Elsevier; 2015. p. 1–25. 10.1016/B978-1-893997-94-3.50007-6.

[14] Hummel J, Segu S, Li Y, Irgang S, Jueppner J, Giavalisco P. Ultra performance liquid chromatography and high resolution mass spectrometry for the analysis of plant lipids. Front Plant Sci. 2011;2:54. 10.3389/fpls.2011.00054 PMC3355513 22629264

[15] Li L, Lu X, Zhao J, Zhang J, Zhao Y, Zhao C, Xu G (2015). Lipidome and metabolome analysis of fresh tobacco leaves in different geographical regions using liquid chromatography–mass spectrometry. Anal Bioanal Chem.

[16] Cenzano AM, Cantoro R, Teresa Hernandez-Sotomayor SM, Abdala GI, Racagni GE (2012). Lipid profiling by electrospray ionization tandem mass spectrometry and the identification of lipid phosphorylation by kinases in potato stolons. J Agric Food Chem.

[17] Sugawara T, Duan J, Aida K, Tsuduki T, Hirata T (2010). Identification of glucosylceramides containing Sphingatrienine in maize and Rice using ion trap mass spectrometry. Lipids..

[18] Li M, Butka E, Wang X. Comprehensive quantification of Triacylglycerols in soybean seeds by electrospray ionization mass spectrometry with multiple neutral loss scans. Sci Rep. 2014;4(1). 10.1038/srep06581. 10.1038/srep06581 PMC4192640 25301200

[19] Boukhchina S, Sebai K, Cherif A, Kallel H, Mayer PM (2004). Identification of glycerophospholipids in rapeseed, olive, almond, and sunflower oils by LCMS and LCMSMS. Can J Chem.

[20] Gao B, Luo Y, Lu W, Liu J, Zhang Y, Yu L (2017). Triacylglycerol compositions of sunflower, corn and soybean oils examined with supercritical CO 2 ultra-performance convergence chromatography combined with quadrupole time-of-flight mass spectrometry. Food Chem.

[21] Leamy LJ, Zhang H, Li C, Chen CY, Song B-H (2017). A genome-wide association study of seed composition traits in wild soybean (Glycine soja). BMC Genomics.

[22] Qu C, Jia L, Fu F, Zhao H, Lu K, Wei L, Xu X, Liang Y, Li S, Wang R, Li J (2017). Genome-wide association mapping and identification of candidate genes for fatty acid composition in Brassica napus L. using SNP markers. BMC Genomics.

[23] Cadic E, Coque M, Vear F, Grezes-Besset B, Pauquet J, Piquemal J (2013). Combined linkage and association mapping of flowering time in Sunflower (*Helianthus annuus* L.). TAG Theor Appl Genet Theor Angew Genet.

[24] Bonnafous F, Fievet G, Blanchet N, Boniface M-C, Carrère S, Gouzy J, Legrand L, Marage G, Bret-Mestries E, Munos S, Pouilly N, Vincourt P, Langlade N, Mangin B (2018). Comparison of GWAS models to identify non-additive genetic control of flowering time in sunflower hybrids. TAG Theor Appl Genet Theor Angew Genet.

[25] Mandel JR, Nambeesan S, Bowers JE, Marek LF, Ebert D, Rieseberg LH, Knapp SJ, Burke JM (2013). Association mapping and the genomic consequences of selection in sunflower. PLoS Genet.

[26] Goryunov DV, Anisimova IN, Gavrilova VA, Chernova AI, Sotnikova EA, Martynova EU, Boldyrev S, Ayupova A, Gubaev R, Mazin P, Gurchenko E, Shumskiy A, Petrova D, Garkusha S, Mukhina Z, Benko N, Demurin Y, Khaitovich P, Goryunova S (2019). Association mapping of fertility restorer gene for CMS PET1 in sunflower. Agronomy..

[27] Masalia RR, Temme AA, de Leon Torralba N, Burke JM (2018). Multiple genomic regions influence root morphology and seedling growth in cultivated sunflower (*Helianthus annuus* L.) under well-watered and water-limited conditions. PloS One.

[28] Mangin B, Casadebaig P, Cadic E, Blanchet N, Boniface M-C, Carrère S, Gouzy J, Legrand L, Mayjonade B, Pouilly N, André T, Coque M, Piquemal J, Laporte M, Vincourt P, Muños S, Langlade NB (2017). Genetic control of plasticity of oil yield for combined abiotic stresses using a joint approach of crop modelling and genome-wide association. Plant Cell Environ.

[29] Nambeesan SU, Mandel JR, Bowers JE, Marek LF, Ebert D, Corbi J, Rieseberg LH, Knapp SJ, Burke JM (2015). Association mapping in sunflower (Helianthus annuus L.) reveals independent control of apical vs. basal branching. BMC Plant Biol.

[30] Dowell JA, Reynolds EC, Pliakas TP, Mandel JR, Burke JM, Donovan LA, Mason CM (2019). Genome-wide association mapping of floral traits in cultivated sunflower (Helianthus annuus). J Hered.

[31] Badouin H, Gouzy J, Grassa CJ, Murat F, Staton SE, Cottret L, Lelandais-Brière C, Owens GL, Carrère S, Mayjonade B, Legrand L, Gill N, Kane NC, Bowers JE, Hubner S, Bellec A, Bérard A, Bergès H, Blanchet N, Boniface MC, Brunel D, Catrice O, Chaidir N, Claudel C, Donnadieu C, Faraut T, Fievet G, Helmstetter N, King M, Knapp SJ, Lai Z, le Paslier MC, Lippi Y, Lorenzon L, Mandel JR, Marage G, Marchand G, Marquand E, Bret-Mestries E, Morien E, Nambeesan S, Nguyen T, Pegot-Espagnet P, Pouilly N, Raftis F, Sallet E, Schiex T, Thomas J, Vandecasteele C, Varès D, Vear F, Vautrin S, Crespi M, Mangin B, Burke JM, Salse J, Muños S, Vincourt P, Rieseberg LH, Langlade NB (2017). The sunflower genome provides insights into oil metabolism, flowering and Asterid evolution. Nature..

[32] Riedelsheimer C, Lisec J, Czedik-Eysenberg A, Sulpice R, Flis A, Grieder C, Altmann T, Stitt M, Willmitzer L, Melchinger AE (2012). Genome-wide association mapping of leaf metabolic profiles for dissecting complex traits in maize. Proc Natl Acad Sci.

[33] Ebrahimi A, Maury P, Berger M, Kiani SP, Nabipour A, Shariati F (2008). QTL mapping of seed-quality traits in sunflower recombinant inbred lines under different water regimes. Genome..

[34] Pérez-Vich B, Fernández-Martínez JM, Grondona M, Knapp SJ, Berry ST (2002). Stearoyl-ACP and oleoyl-PC desaturase genes cosegregate with quantitative trait loci underlying high stearic and high oleic acid mutant phenotypes in sunflower. Theor Appl Genet.

[35] Pérez-Vich B, del Moral L, Velasco L, Bushman BS, Knapp SJ, Leon A, Fernández-Martínez JM, Berry ST (2016). Molecular basis of the high-palmitic acid trait in sunflower seed oil. Mol Breed.

[36] Mangin B, Bonnafous F, Blanchet N, Boniface M-C, Bret-Mestries E, Carrère S, et al. Genomic prediction of sunflower hybrids oil content. Front Plant Sci. 2017;8. 10.3389/fpls.2017.01633. 10.3389/fpls.2017.01633 PMC5613134 28983306

[37] Voelker TA, Kinney AJ (2001). Variations in the biosynthesis of seed-storage lipids. Annu Rev Plant Physiol Plant Mol Biol.

[38] Gavrilova VA, Rozhkova VT, Anisimova IN (2014). Sunflower genetic collection at the Vavilov Institute of Plant Industry. Helia..

[39] Hübner S, Bercovich N, Todesco M, Mandel JR, Odenheimer J, Ziegler E, Lee JS, Baute GJ, Owens GL, Grassa CJ, Ebert DP, Ostevik KL, Moyers BT, Yakimowski S, Masalia RR, Gao L, Ćalić I, Bowers JE, Kane NC, Swanevelder DZH, Kubach T, Muños S, Langlade NB, Burke JM, Rieseberg LH (2019). Sunflower pan-genome analysis shows that hybridization altered gene content and disease resistance. Nat Plants.

[40] Seiler GJ, Gulya TJ, Kong G (2010). Oil concentration and fatty acid profile of wild Helianthus species from the southeastern United States. Ind Crop Prod.

[41] Radanović A, Miladinović D, Cvejić S, Jocković M, Jocić S. Sunflower genetics from ancestors to modern hybrids—a review. Genes. 2018;9(11). 10.3390/genes9110528. 10.3390/genes9110528 PMC6265698 30380768

[42] Salas JJ, Martínez-Force E, Garcés R (2005). Very long chain fatty acid synthesis in sunflower kernels. J Agric Food Chem.

[43] Premnath A, Narayana M, Ramakrishnan C, Kuppusamy S, Chockalingam V. Mapping quantitative trait loci controlling oil content, oleic acid and linoleic acid content in sunflower (*Helianthus annuus* L.). Mol Breed. 2016;36. 10.1007/s11032-016-0527-2.

[44] Merah O, Langlade N, Alignan M, Roche J, Pouilly N, Lippi Y, Vear F, Cerny M, Bouniols A, Mouloungui Z, Vincourt P (2012). Genetic analysis of phytosterol content in sunflower seeds. TAG Theor Appl Genet Theor Angew Genet..

[45] Vanhanen S, West M, Kroon JTM, Lindner N, Casey J, Cheng Q, Elborough KM, Slabas AR (2000). A consensus sequence for long-chain fatty-acid alcohol oxidases from Candida identifies a family of genes involved in lipid ω-oxidation in yeast with homologues in plants and bacteria. J Biol Chem.

[46] Miladinović D, Hladni N, Radanović A, Jocić S, Cvejić S, Kole C (2019). Sunflower and climate change: possibilities of adaptation through breeding and genomic selection. Genomic designing of climate-smart oilseed crops.

[47] Gavrilova VA, Anisimova IN (2017). Genealogy of the sunflower lines created on the basis of Russian varieties. Helia..

[48] Zhigunov AV, Ulianich PS, Lebedeva MV, Chang PL, Nuzhdin SV, Potokina EK (2017). Development of F1 hybrid population and the high-density linkage map for European aspen (Populus tremula L.) using RADseq technology. BMC Plant Biol.

[49] *Helianthus annuus* (ID 620114) - BioProject - NCBI. https://www.ncbi.nlm.nih.gov/bioproject/620114. Accessed 21 Oct 2020.

[50] Li H, Durbin R (2009). Fast and accurate short read alignment with burrows-wheeler transform. Bioinforma Oxf Engl.

[51] Purcell S, Neale B, Todd-Brown K, Thomas L, Ferreira MAR, Bender D, Maller J, Sklar P, de Bakker PIW, Daly MJ, Sham PC (2007). PLINK: a tool set for whole-genome association and population-based linkage analyses. Am J Hum Genet.

[52] Alexander DH, Novembre J, Lange K (2009). Fast model-based estimation of ancestry in unrelated individuals. Genome Res.

[53] Danecek P, Auton A, Abecasis G, Albers CA, Banks E, DePristo MA (2011). The variant call format and VCFtools. Bioinformatics..

[54] Giavalisco P, Li Y, Matthes A, Eckhardt A, Hubberten H-M, Hesse H, et al. Elemental formula annotation of polar and lipophilic metabolites using 13C, 15N and 34S isotope labelling, in combination with high-resolution mass spectrometry. Plant J. 2011;68. 10.1111/j.1365-313X.2011.04682.x. 10.1111/j.1365-313X.2011.04682.x 21699588

[55] Bromke MA, Hochmuth A, Tohge T, Fernie AR, Giavalisco P, Burgos A, Willmitzer L, Brotman Y (2015). Liquid chromatography high-resolution mass spectrometry for fatty acid profiling. Plant J.

[56] Chernova A, Mazin P, Goryunova S, Goryunov D, Demurin Y, Gorlova L, Vanyushkina A, Mair W, Anikanov N, Yushina E, Pavlova A, Martynova E, Garkusha S, Mukhina Z, Savenko E, Khaitovich P (2019). Ultra-performance liquid chromatography-mass spectrometry for precise fatty acid profiling of oilseed crops. PeerJ..

[57] Chernova A, Gubaev R, Mazin P, Goryunova S, Demurin Y, Gorlova L, et al. UPLC^−^MS triglyceride profiling in sunflower and rapeseed seeds. Biomolecules. 2018;9(1). 10.3390/biom9010009. 10.3390/biom9010009 PMC6359410 30591683

[58] Smith CA, Want EJ, O’Maille G, Abagyan R, Siuzdak G (2006). XCMS: processing mass spectrometry data for metabolite profiling using nonlinear peak alignment, matching, and identification. Anal Chem.

[59] Bradbury PJ, Zhang Z, Kroon DE, Casstevens TM, Ramdoss Y, Buckler ES (2007). TASSEL: software for association mapping of complex traits in diverse samples. Bioinformatics..

[60] Barrett JC, Fry B, Maller J, Daly MJ (2005). Haploview: analysis and visualization of LD and haplotype maps. Bioinforma Oxf Engl..

