# Supplementary figures and images for: Genotyping and lipid profiling of 601 cultivated sunflower lines reveals novel genetic determinants of oil fatty acid content
Source: BMC Genomics. 2021 Jul 5;22:505. doi: 10.1186/s12864-021-07768-y (PMC8256595; doi:10.1186/s12864-021-07768-y)

A

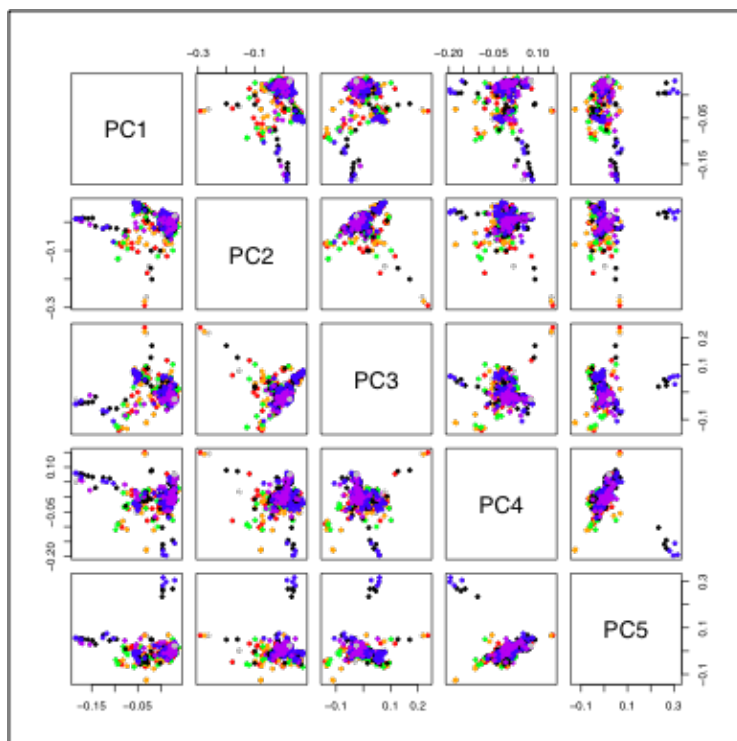

B

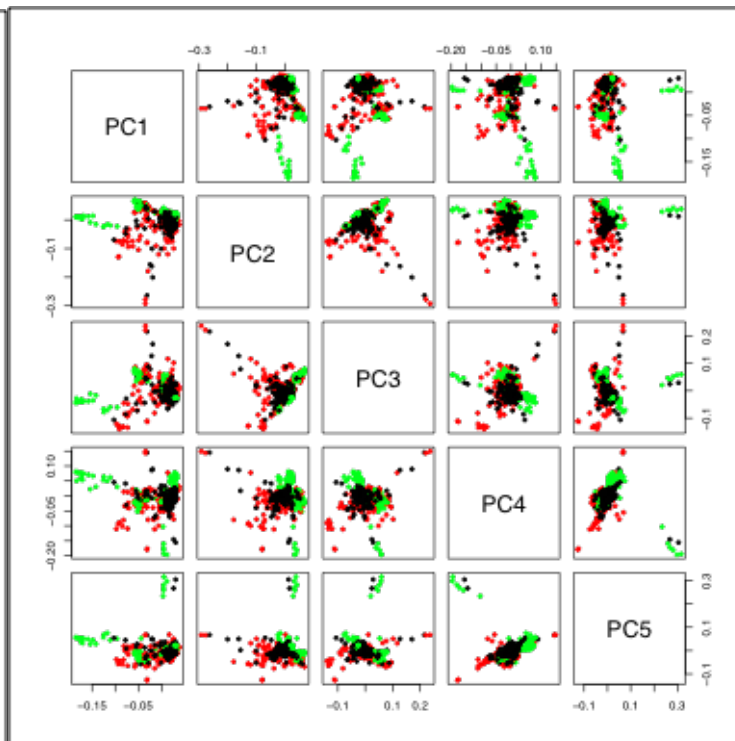

C

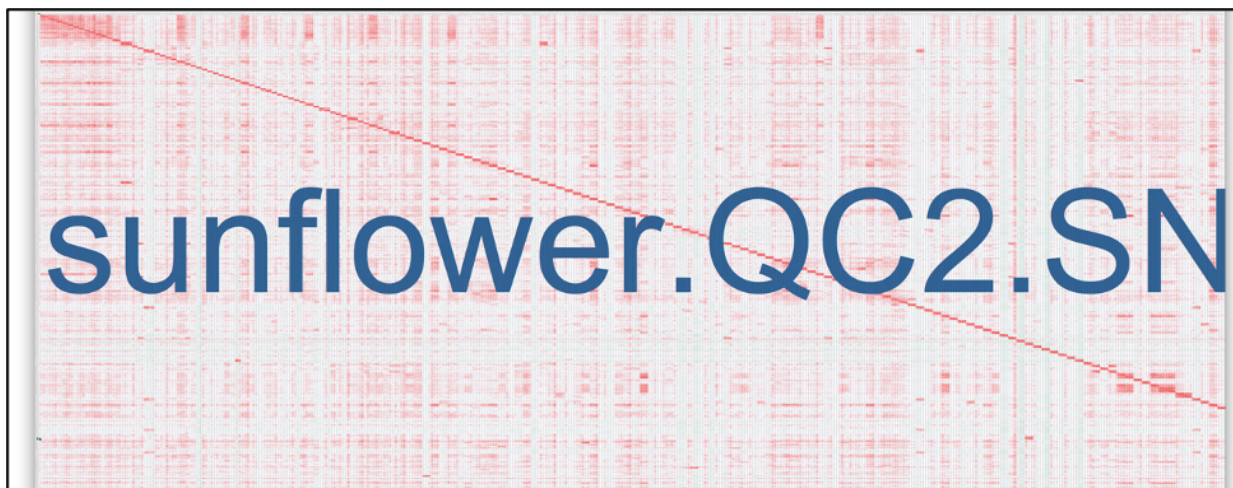

Supplement: Supplementary file 1 — Additional file 1: FigS1. ((A) PCA plots reflecting the relationships between sunflower technical samples based on 15,068 SNPs segregating in the Russian collection. Each dot corresponds to a sunflower technical sample used in the study. Dots are colored by sequencing batch (A) or collection samples were obtained from (B) (C) Nei’s genetic distance matrix between each 2 samples. [file 12864_2021_7768_MOESM1_ESM.pdf]

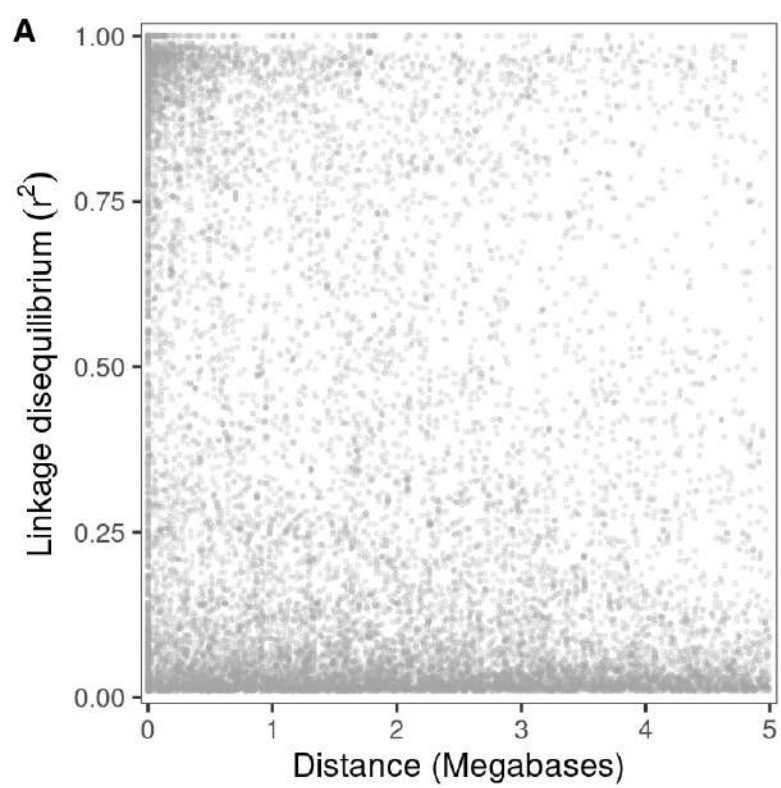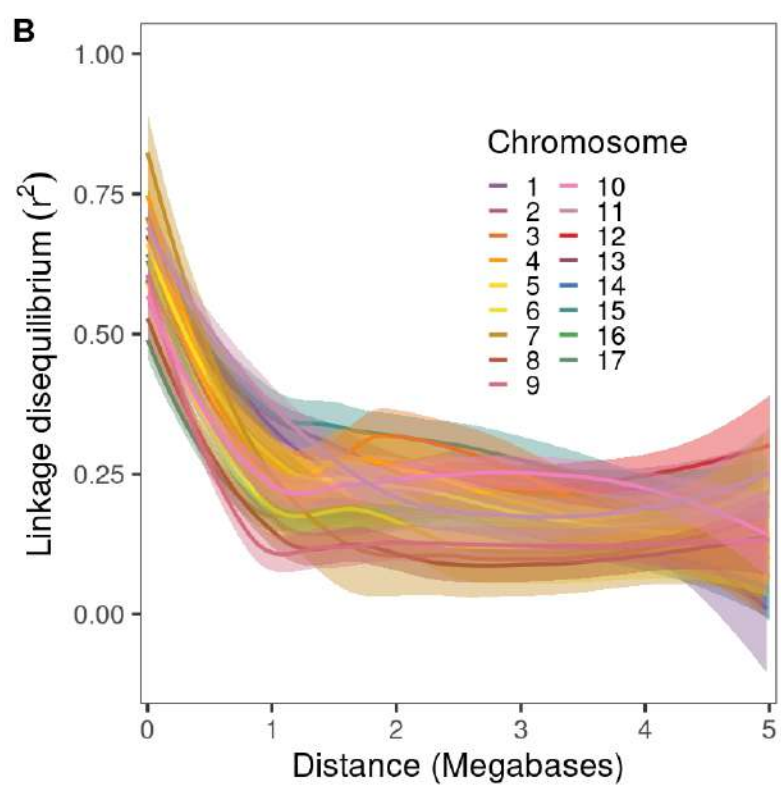

Supplement: Supplementary file 2 — Additional file 2: FigureS2. Linkage disequilibrium (LD) decay plot. (A) Genome-wide LD. Gray dots correspond to a SNP pair, y-axis show r2 between two SNPs calculated using whole dataset. SNP pairs with distance less than 5 Mb are shown. (B) LD per each chromosome. Lines correspond to loess curves; 95% confidence intervals are shown by shades. [file 12864_2021_7768_MOESM2_ESM.pdf]

**A**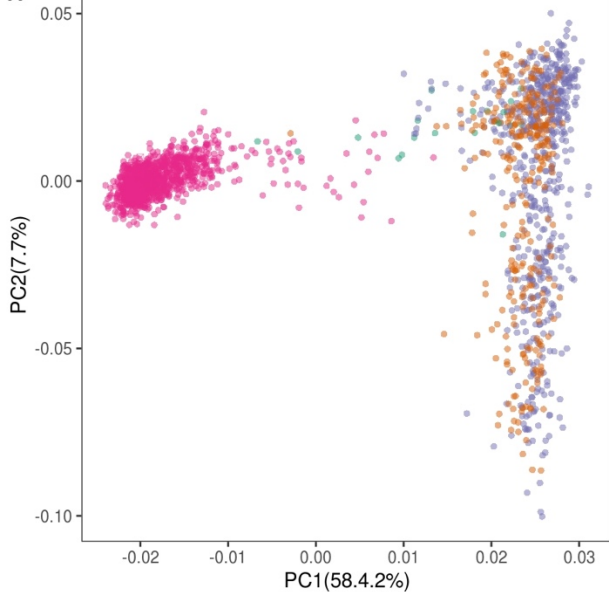**B**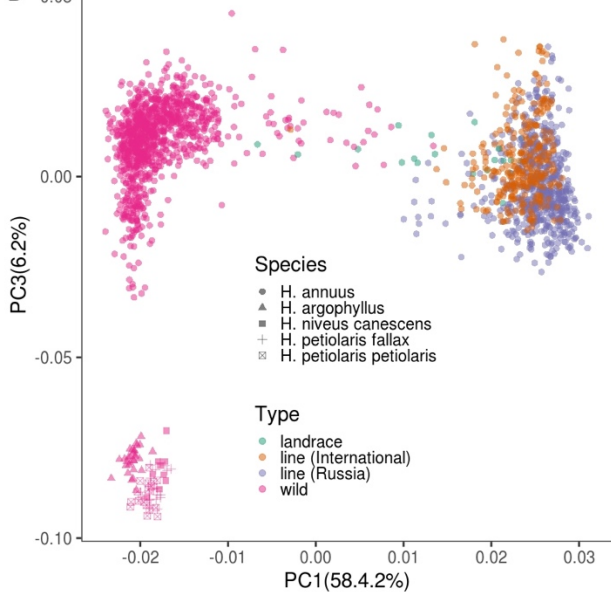

Supplement: Supplementary file 3 — Additional file 3: FigureS3. Joint principal component analysis of sunflower accessions genotyped in this study and in Hübner (2019) based on 2345 shared SNPs. The first and the second (A) or the first and the third (B) PCs are shown. Each dot corresponds to a plant accession. Colors indicate the origin (wild/line/landrace). Shapes indicate species. [file 12864_2021_7768_MOESM3_ESM.pdf]

**A**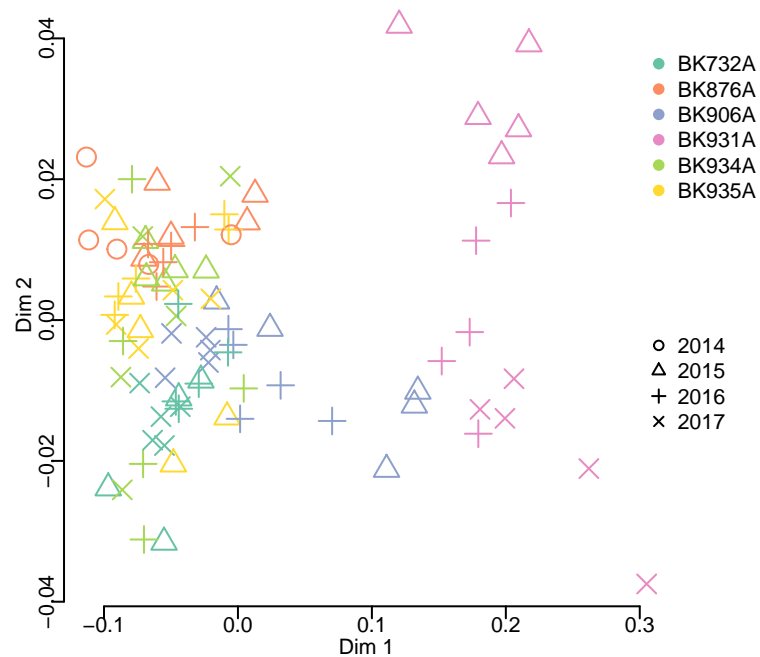**B**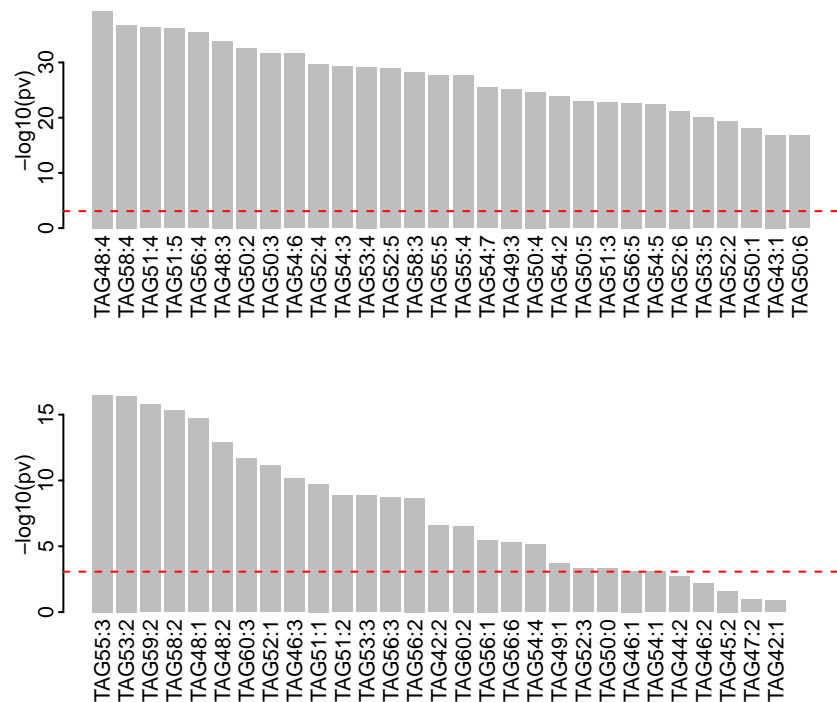**C**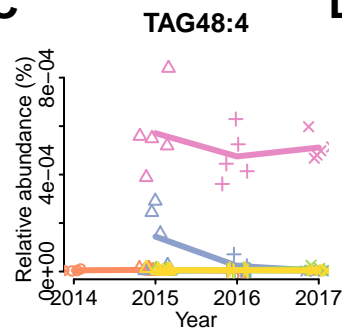**D**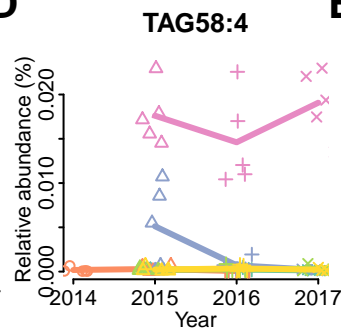**E**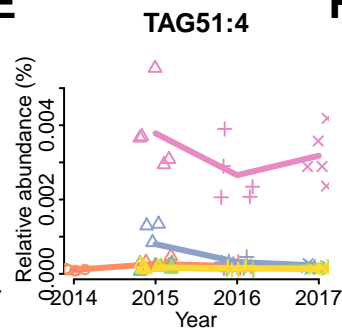**F**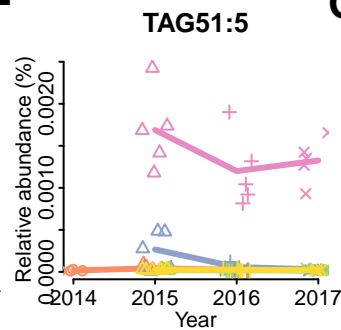**G**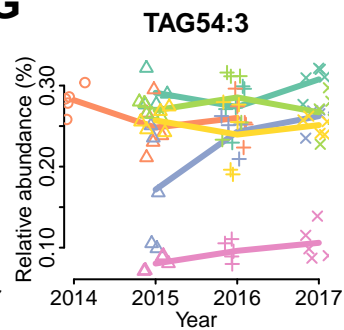

Supplement: Supplementary file 4 — Additional file 4 (A) Multidimensional scaling plot (two dimensions, 1 - Spearman correlation coefficient between TAGs abundances measured using LC-MS with dilution 1:3 was used as distance). One sample is shown by one point; accessions are shown by different colors; different years are shown by points of different shapes. (B) Minus log10 p-values for the differences between lines (ANOVA) are shown, TAGs are ordered by p-value increase from top left to bottom right. Bonferroni adjusted 0.05 significance level is shown by red line; (C-G) abundances of five selected TAGs are shown across lines and years. Each point represents 1 sample, point shapes, and colors as in (A), lines show per-year averages. This figure complements main Fig. 3. [file 12864_2021_7768_MOESM4_ESM.pdf]

**A**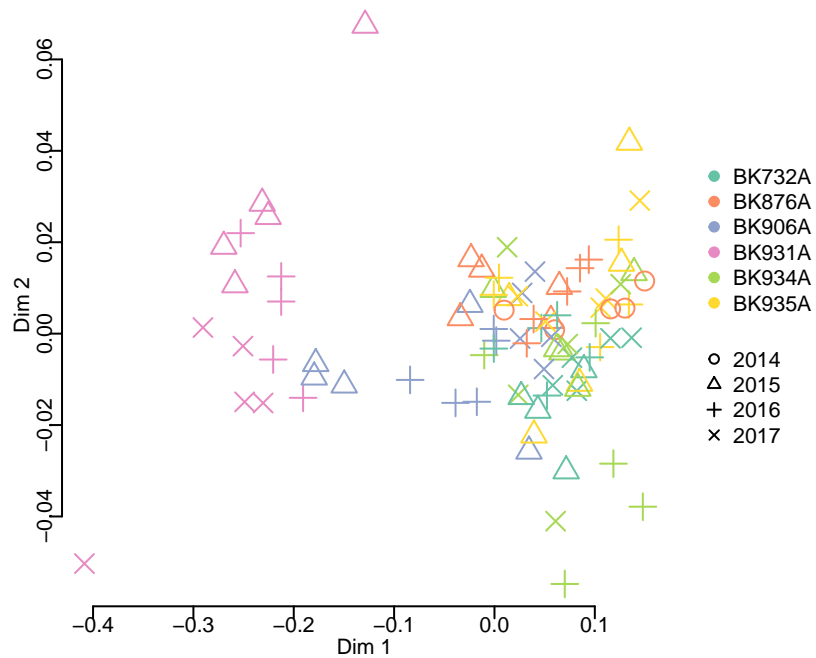**B**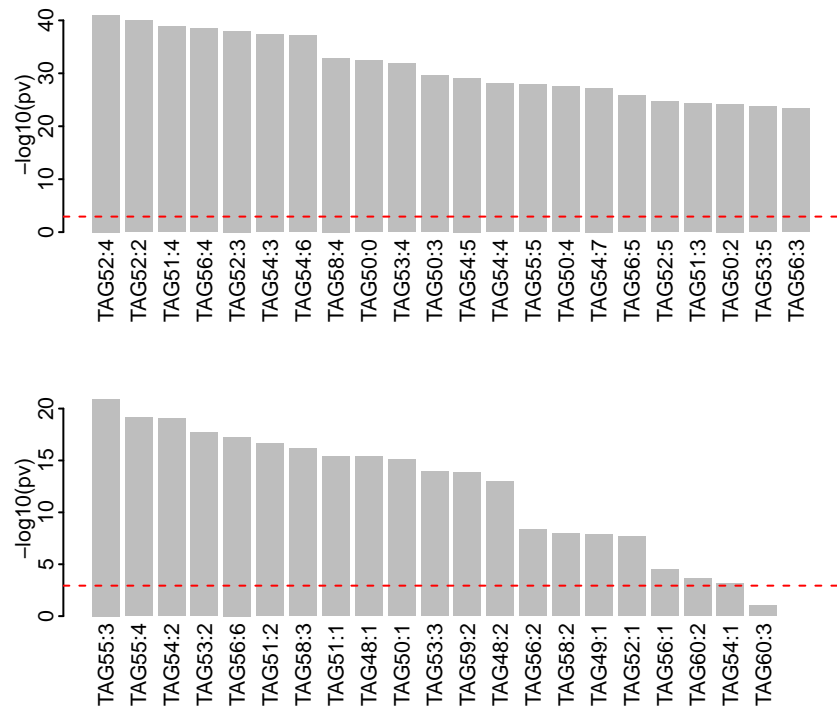**C**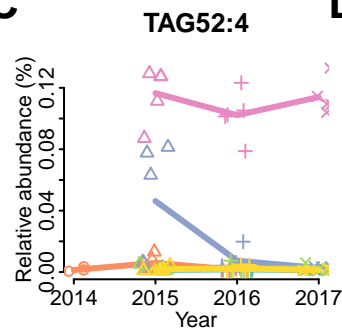**D**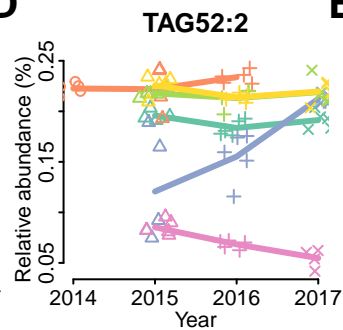**E**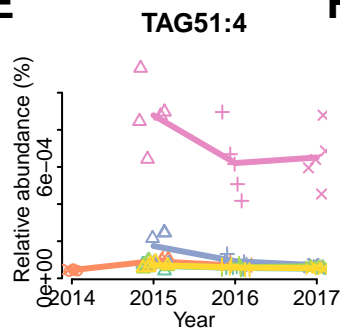**F**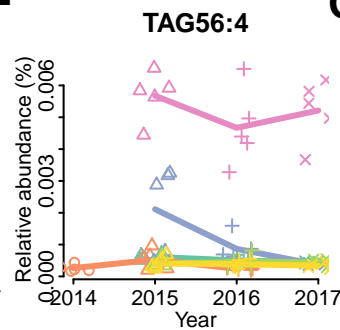**G**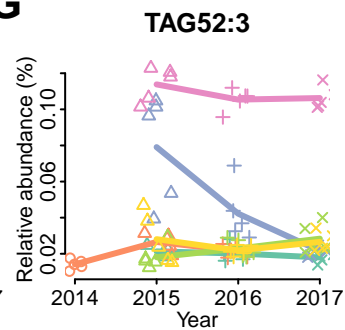

Supplement: Supplementary file 5 — Additional file 5: FigureS5. (A) Multidimensional scaling plot (two dimensions, 1 - Spearman correlation coefficient between TAGs abundances measured using LC-MS with dilution 1:25 was used as distance). One sample is shown by one point; accessions are shown by different colors; different years are shown by points of different shapes. (B) Minus log10 p-values for the differences between lines (ANOVA) are shown, TAGs are ordered by p-value increase from top left to bottom right. Bonferroni adjusted 0.05 significance level is shown by red line; (C-G) abundances of five selected TAGs are shown across lines and years. Each point represents 1 sample, point shapes, and colors as in (A), lines show per-year averages. This figure complements main Fig. 3. [file 12864_2021_7768_MOESM5_ESM.pdf]

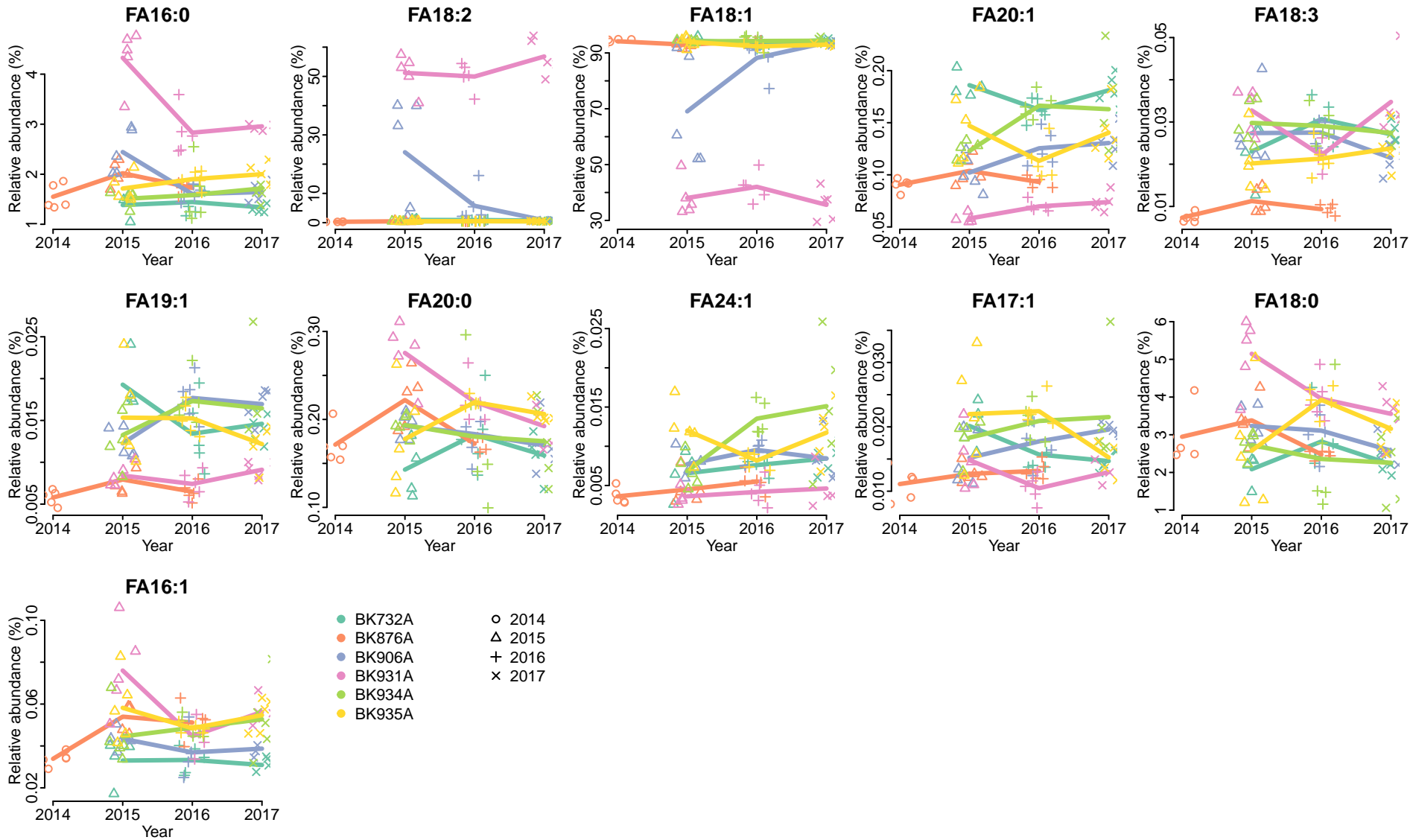

Supplement: Supplementary file 6 — Additional file 6: FigureS6. Replication experiment on 6 accessions: relative abundances of all the fatty acids with significant (FDR < 0.05) effect of genotype, FAs are ordered by p-value increase. Each point represents 1 sample, point shapes, and colors denote years and accessions, respectively, lines show per-year averages. [file 12864_2021_7768_MOESM6_ESM.pdf]

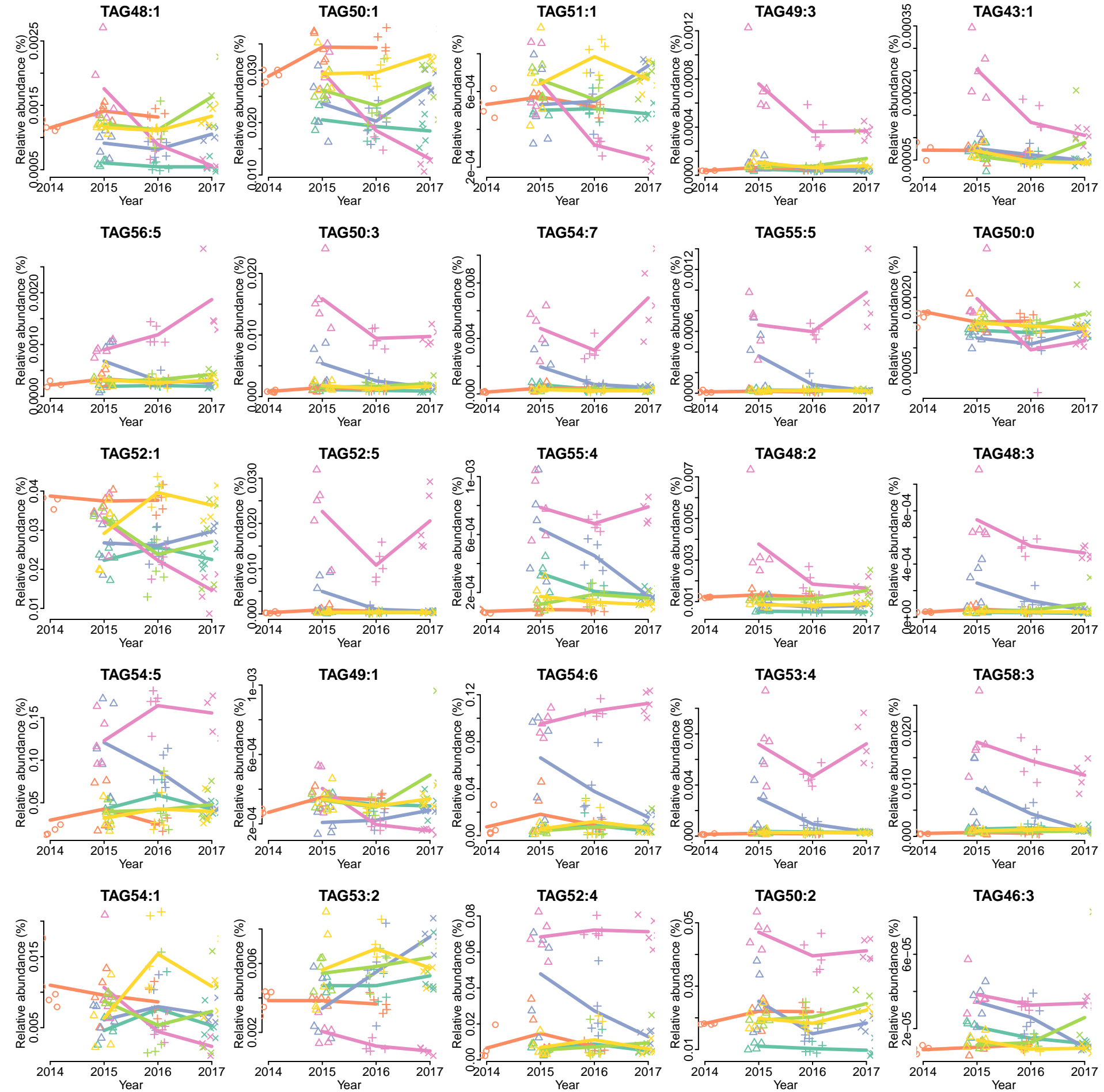

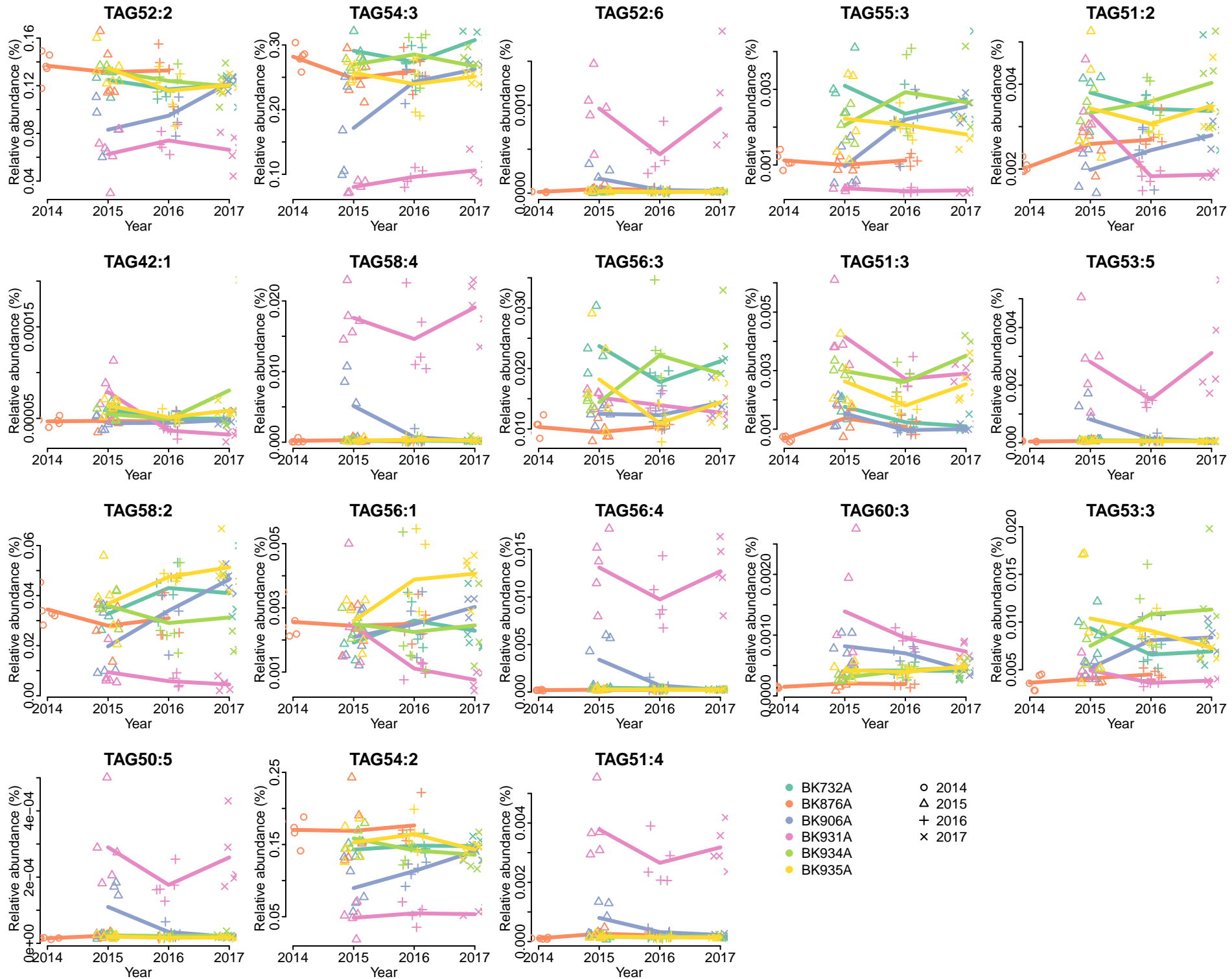

Supplement: Supplementary file 7 — Additional file 7: FigureS7. Replication experiment on 6 genotypes: relative abundances of all TAGs detected by LC-MS with dilution 1:3 with significant (FDR < 0.05) effect of genotype, TAGs are ordered by p-value increase. Each point represents 1 sample, point shapes, and colors denote years and accessions, respectively, lines show per-year averages. [file 12864_2021_7768_MOESM7_ESM.pdf]

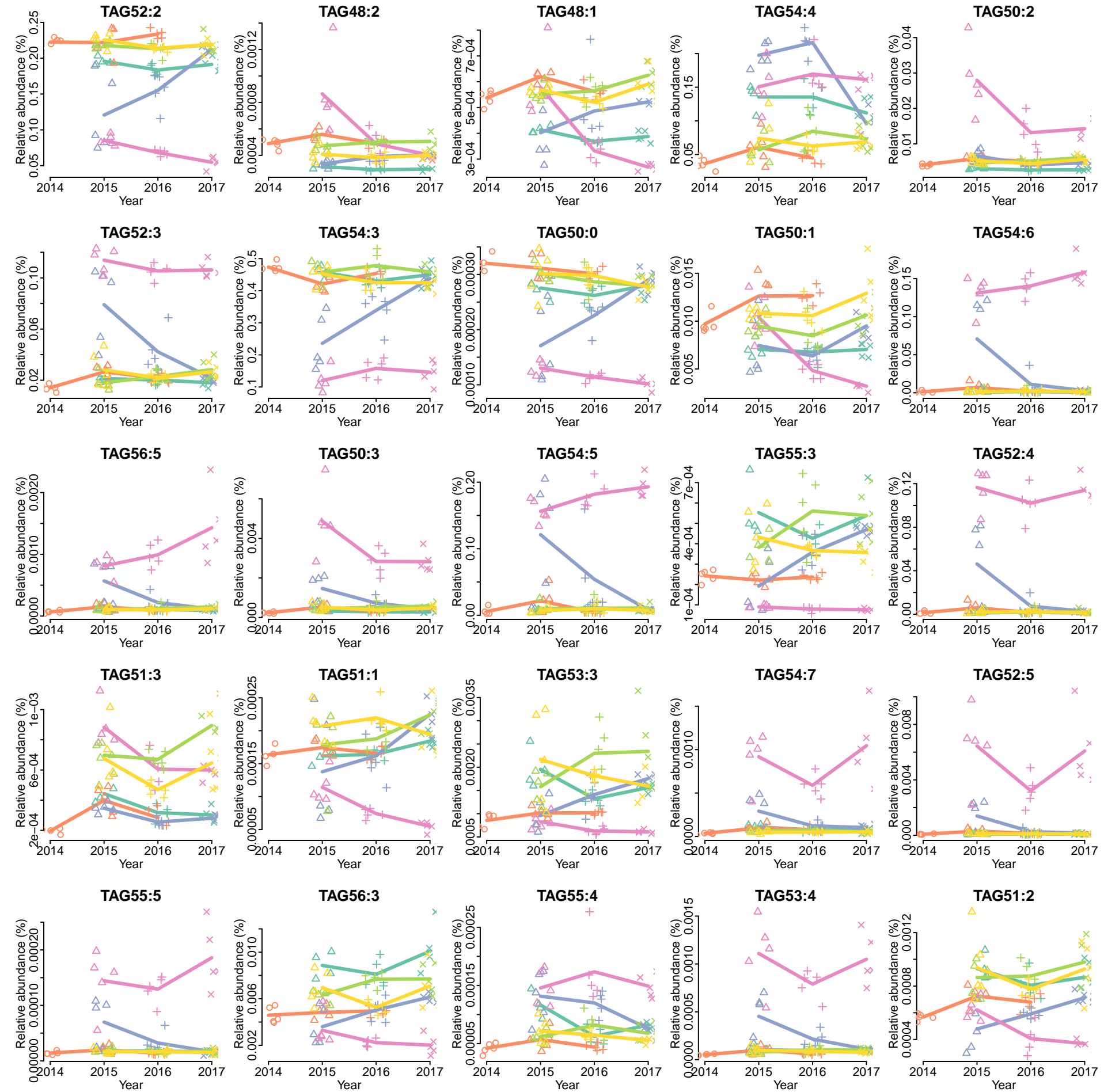

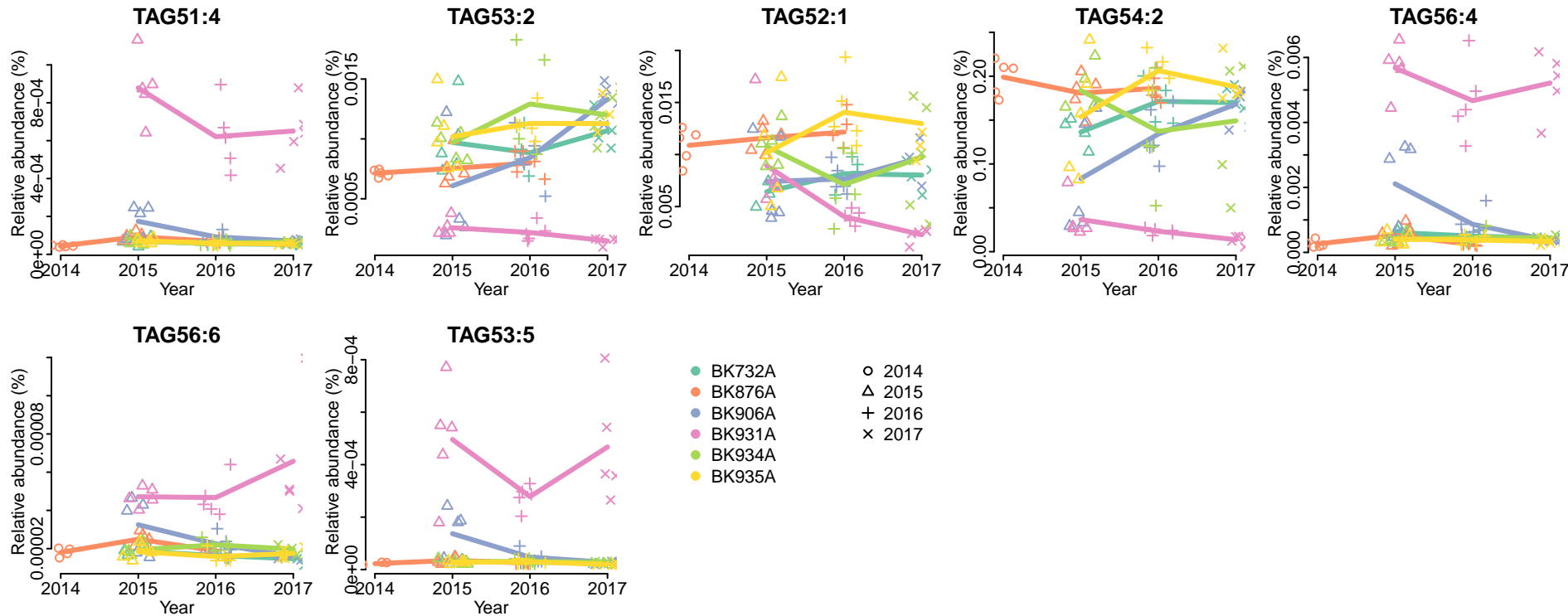

Supplement: Supplementary file 8 — Additional file 8: FigureS8. Replication experiment on 6 genotypes: relative abundances of all TAGs detected by LC-MS with dilution 1:25 with significant (FDR < 0.05) effect of genotype, TAGs are ordered by p-value increase. Each point represents 1 sample, point shapes, and colors denote years and accessions, respectively, lines show per-year averages. [file 12864_2021_7768_MOESM8_ESM.pdf]

**FA**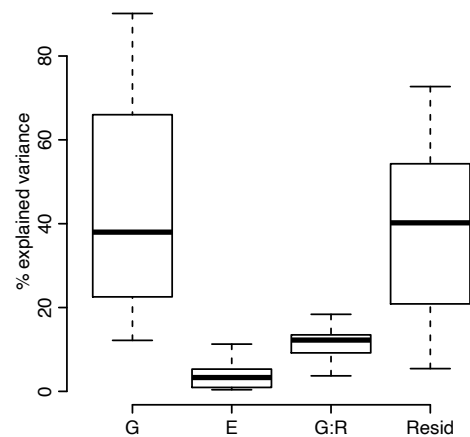**FA**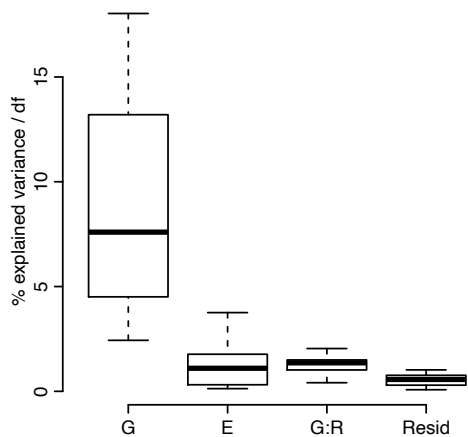**TAG 1:25**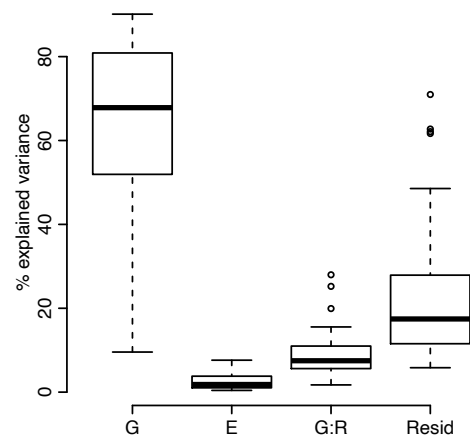**TAG 1:25**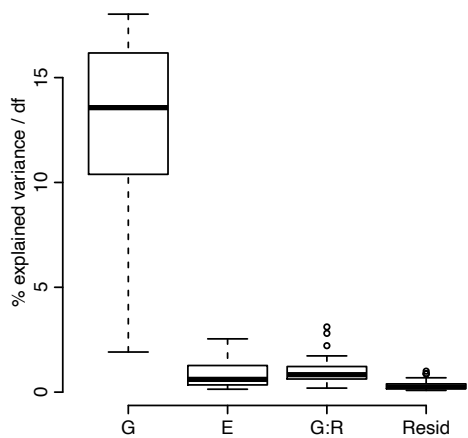**TAG 1:3**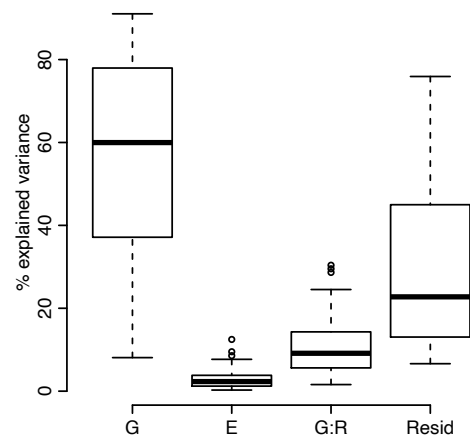**TAG 1:3**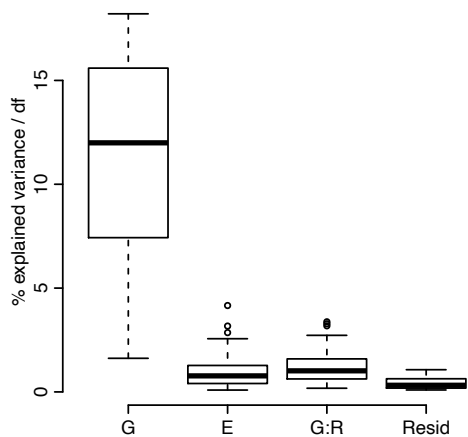

Supplement: Supplementary file 9 — Additional file 9: FigureS9. Replication experiment on 6 accessions. (Left) Distributions of FAs (top) or TAGs (middle and bottom) by percentages of variance explained by genotype (G), environment (year, E), interaction between genotype and environment (G:E) or percentage of residual variance. Distribution of the same percentages divided by number of degrees of freedom of corresponding factor are shown on the right. [file 12864_2021_7768_MOESM9_ESM.pdf]

18:1 Chr6

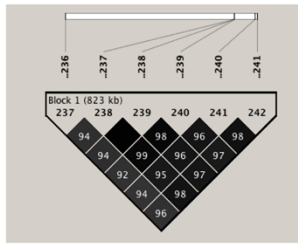

18:1 Chr9

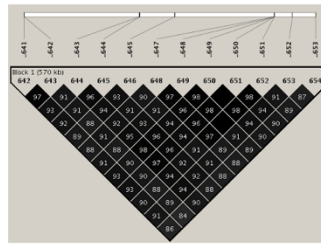

18:1 Chr13

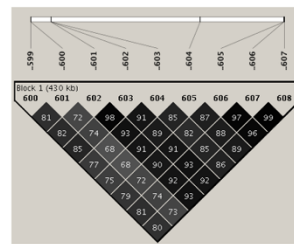

18:1 Chr15

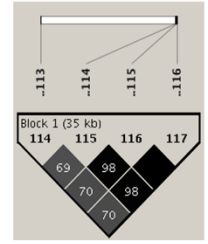

18:2 Chr3

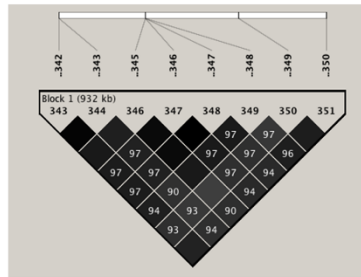

18:2 Chr5

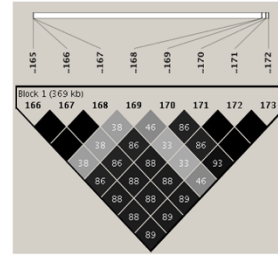

18:2 Chr11

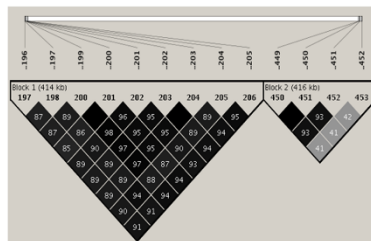

18:3 Chr11

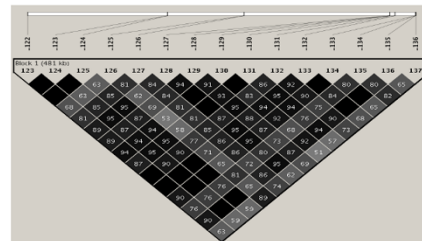

19:0 Chr2

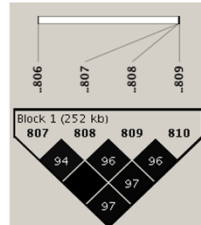

19:0 Chr14

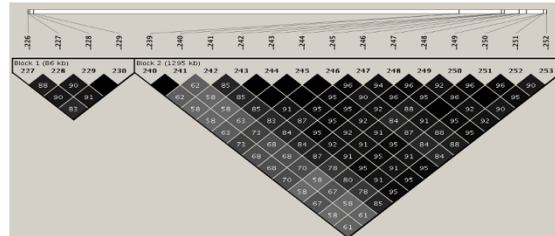

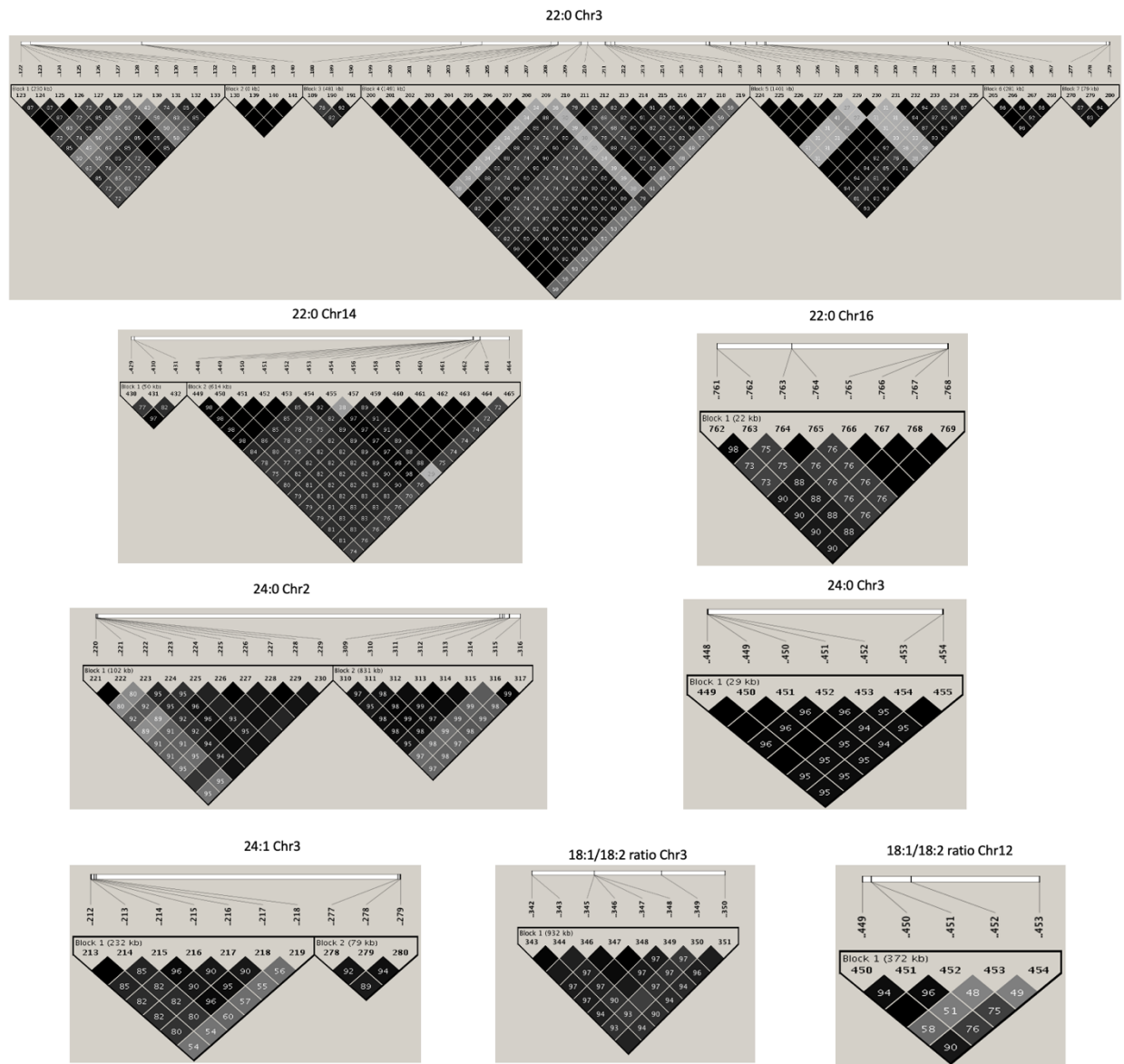

Figure S11: LD blocks containing significant SNPs.

Supplement: Supplementary file 11 — Additional file 11: FigureS11. LD blocks containing significant SNPs. Each panel represents one significant association, traits and chromosome names where significant loci is located are shown in panel titles. Each panel schematically shows associated loci with all detected SNPs. LD-blocks detected by Haploview software are shown by heatmaps, r2 (%) for each SNP pair is shown by color and numbers. [file 12864_2021_7768_MOESM11_ESM.pdf]

**FA**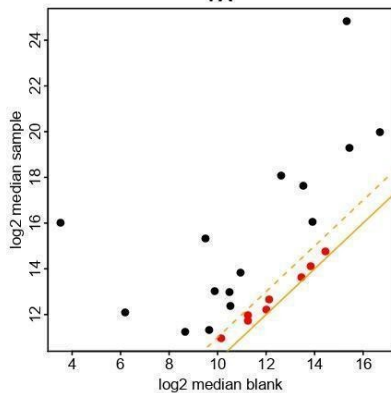**TAG x25**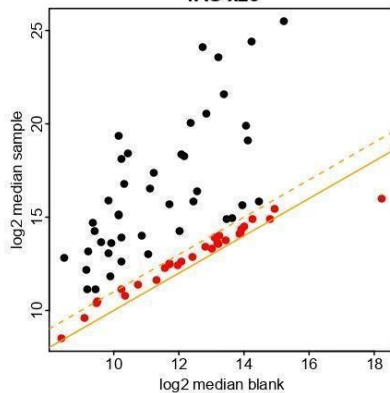**TAG x3**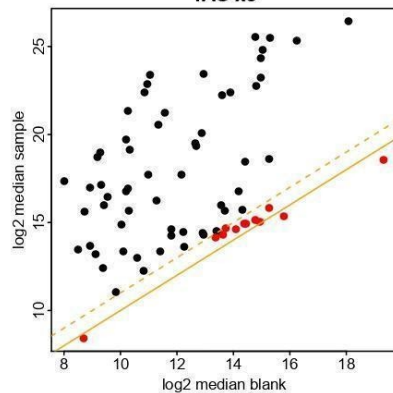**FA**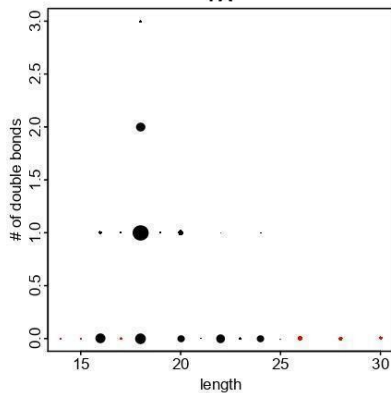**TAG x25**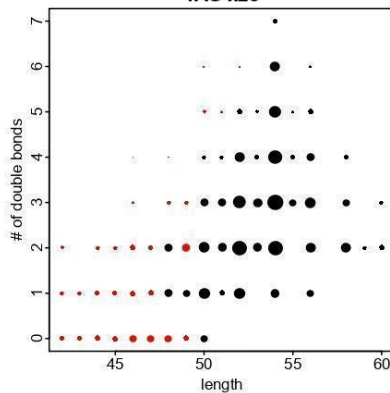**TAG x3**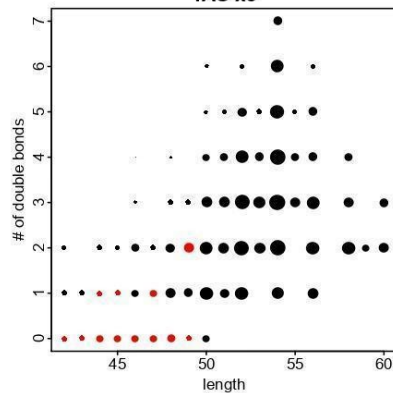

Supplement: Supplementary file 12 — Additional file 12: FigureS12. Data clean up using blank samples. Top panels show dependence of average log2 seed sample intensity of FAs and TAGs (two dilutions) on average log2 intensity of same lipids in blank samples (see Methods). Straight and dashed lines correspond to equal intensities in both types of sample and to two-fold higher concentration in seed samples compared to blanks, respectively. Bottom panels show the same lipids as top panels in coordinates of total FA (TAG) chain length (x-axis), and number of double bounds (y-axis), point size is proportional to log2 average intensity in seed samples. Only lipids with log2(sample/blank) > 1 were used in analysis, remaining FAs and TAGs (shown in red) were filtered out. [file 12864_2021_7768_MOESM12_ESM.pdf]

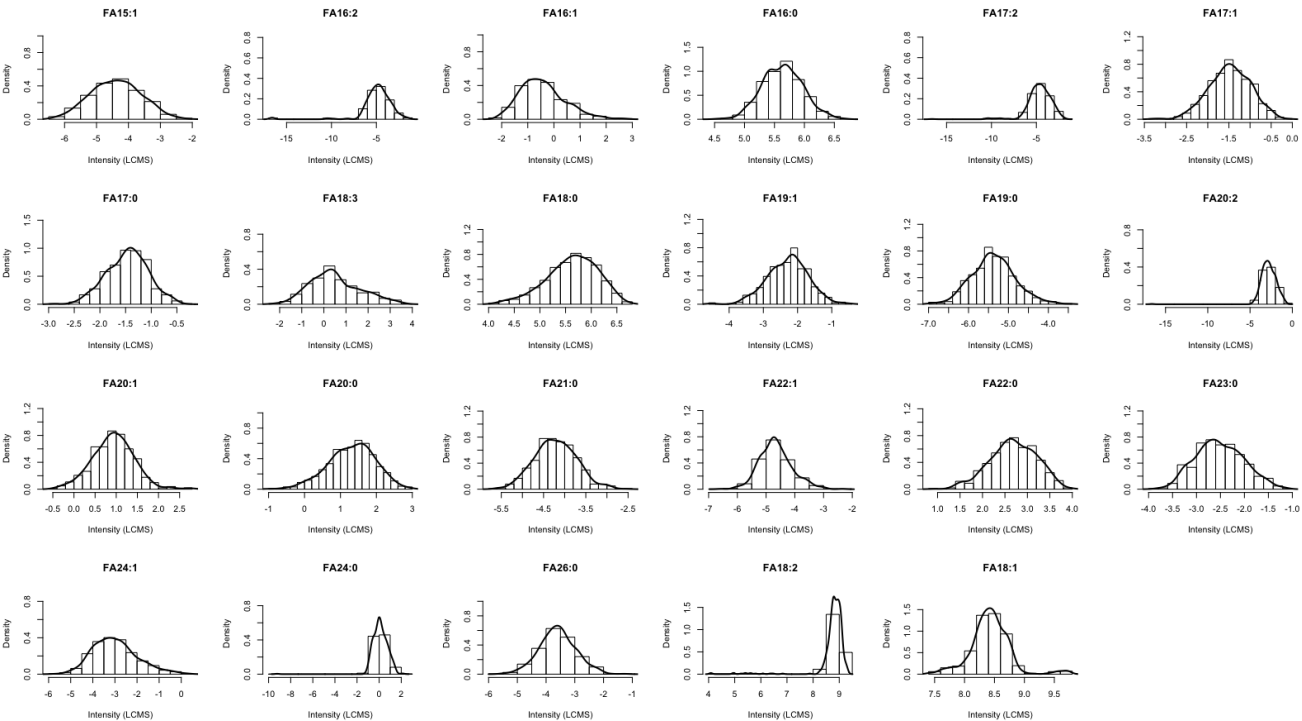

Sample weight normalized and log2 transformed

Supplement: Supplementary file 13 — Additional file 13: FigureS13. Distributions of natural logarithm of row LC-MS fatty acids intensities across accessions. [file 12864_2021_7768_MOESM13_ESM.pdf]
