# Supplementary material for: Genotyping and lipid profiling of 601 cultivated sunflower lines reveals novel genetic determinants of oil fatty acid content
Source: BMC Genomics. 2021 Jul 5;22:505. doi: 10.1186/s12864-021-07768-y (PMC8256595; doi:10.1186/s12864-021-07768-y)
Supplement: Supplementary file 10 — Additional file 10: FigureS10. GWAS Manhattan plots for (a) Stearic acid; (b) Nonadecanoic acid; (c) Eicosenoic acid; (d) Docosanoic acid; (e) Tetracosanoic acid; (f) Nervonic acid; (g) Oleic acid; (h) Linoleic acid; (i) Ratio between oleic and linoleic acids. FDR and Bonferroni thresholds are shown by blue and red, respectively. [file 12864_2021_7768_MOESM10_ESM.pdf]

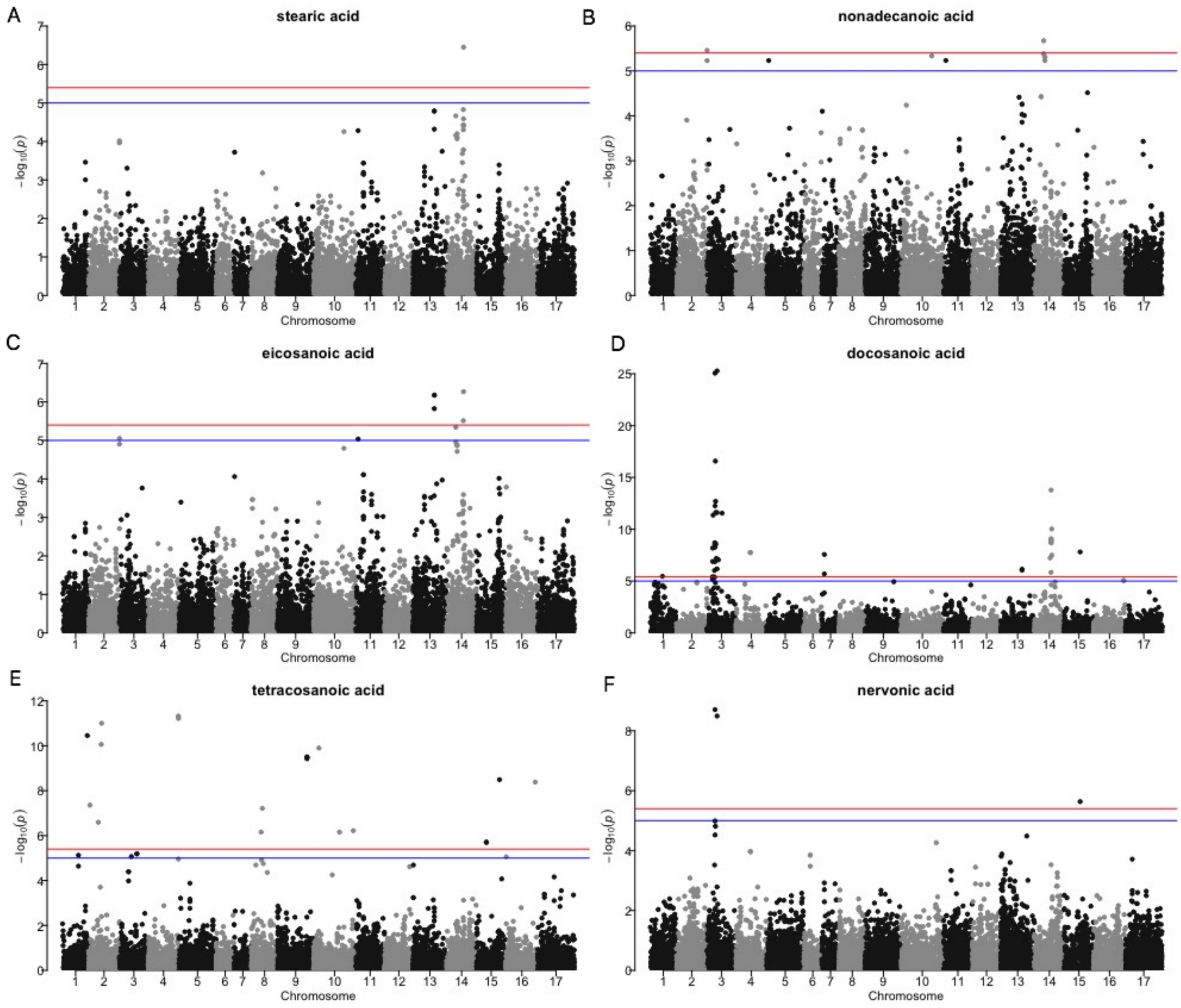

A
stearic acid
B
nonadecanoic acid
C
eicosanoic acid
D
docosanoic acid
E
tetracosanoic acid
F
nervonic acid
Chromosome
-log10(p)

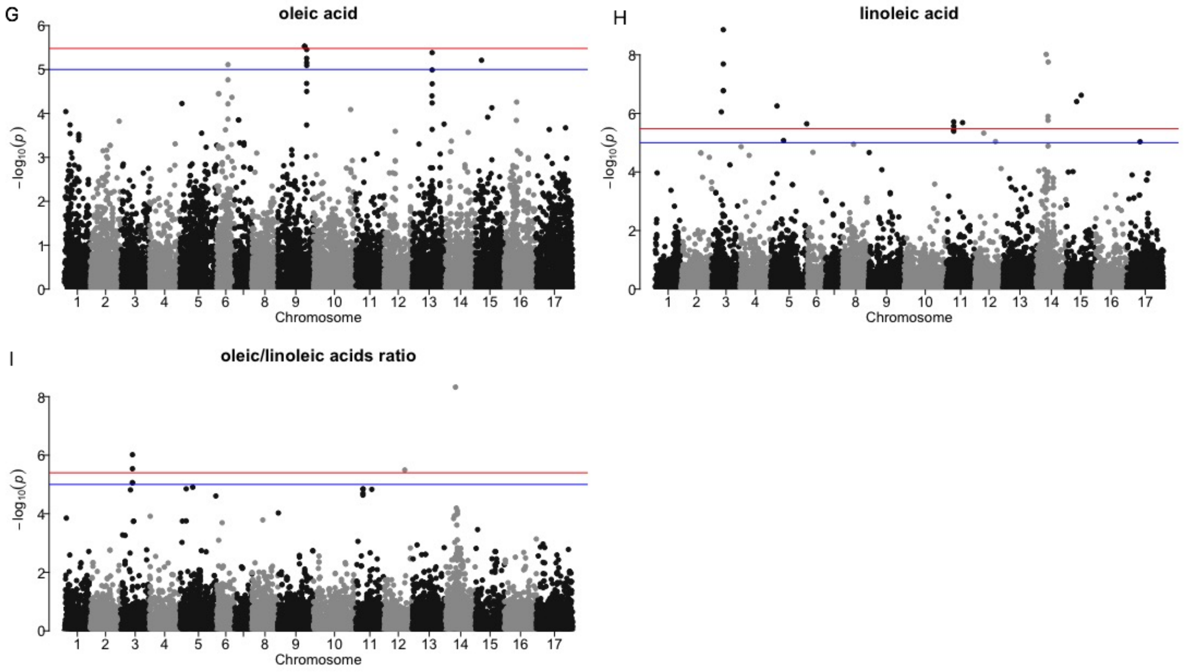

Figure S10: Manhattan plots representing significant associations (a) Stearic acid; (b) Nonadecanoic acid; (c) Eicosenoic acid; (d) Docosanoic acid; (e) Tetracosanoic acid; (f) Nervonic acid; (g) Oleic acid; (h) Linoleic acid; (i) Ratio between oleic and linoleic acids. FDR threshold - blue, Bonferroni threshold -red.
