# Supplementary material for: Genotyping and lipid profiling of 601 cultivated sunflower lines reveals novel genetic determinants of oil fatty acid content
Source: BMC Genomics. 2021 Jul 5;22:505. doi: 10.1186/s12864-021-07768-y (PMC8256595; doi:10.1186/s12864-021-07768-y)
Supplement: Supplementary file 22 — Additional file 22: Supporting experimental procedures. Method S1. Reagents, Method S2. GBS library preparation, Method S3. Lipid extraction and UPLC-MS. [file 12864_2021_7768_MOESM22_ESM.docx]

Supporting experimental procedures

Methods S1

Reagents

NucleoSpin® Plant II plant DNA extraction kit (Macherey-Nagel), AMPureXP (Beckman Coulter A63881), Qubit dsDNA High-Sensitivity Assay Kit (Life Technologies Q32854), Agarose I (Amresco Amresco ), SafeView (abm G108), EDTA (0.5M), Buffer EB (Qiagen 19086), polyethylene glycol 8000 solution (Sigma-Aldrich P5413 ), MgCl2, HindIII (20,000U/mL, NEB #R0104S), NlaIII(10,000U/mL, 2500U NEB #R0125), 10x NEB buffer 2.1 (NEB #B7202), 10x Cut Smart NEB (NEB #B7204), T4 ligase (NEB #A63881), Phusion® High-Fidelity DNA Polymerase (NEB M0530L),Phusion® High-Fidelity PCR Master Mix with HF Buffer (NEB M0531S), Ultrapure 1M Tris-HCL Buffer pH=7.5 (Life Technologies 15567-027),dNTP (10 mM, NEB N0447S), ETOH 95%, Agilent High Sensitivity DNA kit (Agilent),

Methanol LC-MS (Scharlau, Spain), Methyl tert-butyl ether HPLC grade (Scharlau, Spain), Chloroform HPLC grade (Fisher Chemical, USA), Heptane LC/MS grade (Honeywell Fluka, USA), Water UHPLC-MS grade (Scharlau, Spain), Potassium hydroxide solution 45% in water (Sigma Aldrich, USA ), NaCl USP grade (Helicon, Russia) HCl 37% (PanReac AppliChem,USA), Lipid standards (Oleic acid-^13^C_18 (#_490431 Sigma Aldrich_),_ Palmitic acid-^13^C_16 (#_605573 Sigma Aldrich _and_ Stearic acid-^13^C_18 (#_605581 Sigma Aldrich, 18:1-d7, LPC _#_791643 Avanti, and 15:0-18:1-d7 DG _#_791647 Avanti), Acetonitrile LC/MS grade (Fisher Chemical, USA), Isopropanol LC-MS grade (Honeywell Fluka, USA), Ammonium acetate (Honeywell Fluka, USA), Formic acid 98%-100% LC-MS grade (LiChropur Merck Millipore, USA), Acetic acid Optima, LC-MS grade (Fisher Chemical, USA)

Methods S2

GBS library preparation

Before the procedure, DNA concentration for each sample was diluted to 10 ng/uL in 96-well plates (1 well per sample) and working adapter stocks (0.5 mM) were prepared. 2 uL of “barcode” adaptor (5mM) was added into each well. To perform the first restriction digestion with HindIII, Master Mix containing 0.5 uL of HindIII (NEB, USA) with working concentration of 20U/uL, 2 uL of CutSmart buffer (NEB, USA), and 5.5 uL of mQ was prepared. Then, 8 uL of Master Mix was added to each well containing 100 ng of DNA in 10 uL, and digested for 1 hour at 37°C in a thermal cycler (Thermo SimpliAmp). The digestion was terminated by incubation of the reaction mix at 65°C for 20 min. Right after that, the ligation reaction was carried out. The “barcode” adapter was ligated to the sticky ends produced by HindIII, allowing to pool the samples afterward. Master Mix including 1.5 uL of T4 ligase (NEB, USA) with a working concentration of 400U/uL, 5 uL of T4 buffer, and 23.4 uL mQ was prepared. Next, 30 uL of Master Mix was added to each well and incubated at 22°C for 2 hours. Inactivation of T4 ligase was performed by heating at 65°C for 10 min. Then, 10 uL was taken from each sample and pooled in one Eppendorf tube in order to simultaneously purify them using PEG8000 according to instructions provided in a PEG purification protocol. To perform the second restriction digestion with NlaIII, Master Mix including 0.7 uL of NlaIII (NEB, USA) with working concentration of 20U/uL, 2 uL of CutSmart buffer (NEB, USA), and 16.1 uL of mQ+DNA mix was prepared. 2 uL of another adapter (5mM) called “common” 30 was added into each well in order to be ligated to the overhanging ends generated by NlaIII afterward. The digestion was carried out at 37°C for 15 min with the following inactivation at 65°C for 20 min. Then, the ligation reaction was performed. Master Mix consisting of 1.5 uL of T4 ligase (NEB, USA) with a working concentration of 400U/uL, 5 uL of T4 buffer, and 23.4 uL mQ was prepared. After that, 30 uL of Master Mix was added to each well and incubated at 22°C for 2 hours. Inactivation of T4 ligase was performed by heating at 65°C for 10 min. The next step was to clean up the samples using AMPure beads (Agencourt AMPureXP kit) according to the instructions provided by the manufacturer. After purification, PCR reaction was conducted to amplify the DNA fragments. The reaction mixture volume (50 uL) was distributed among 16 aliquots in order to reduce the risk of mistakes during PCR. Master Mix contained 28 uL of mQ, 5 uL of Primer 1 (5uM), 5 uL of Primer 2 (5uM), and 10 uL of Phusion high-fidelity PCR Master Mix with HF buffer, dNTPs, and Phusion high-fidelity DNA polymerase (NEB, USA). PCR conditions were the following: 98°C for 30 sec, 14 cycles of 98°C for 10 sec, 65°C for 30 sec, 72°C for 15 sec, 72°C for 2 min, and then at 4°C. All 16 PCR reactions were separately purified with AMPure beads, and the double selection was conducted. The double selection allowed to remove small and large fragments, so the library mainly consisted of fragments about 400-500 bp. Library concentrations were first checked with Qubit 3.0 Fluorometer (Thermo fisher scientific) and the fragment length distribution was examined with 2100 BioAnalyser (Agilent) following Agilent High Sensitivity DNA Assay Protocol.

Methods S3

Lipid extraction

After homogenization, 400 **u**l more of methanol/methyl tert-butyl ether mixture was added to each sample. Then each sample was vortexed. After sonication for 10 min in an ice-cooled sonic bath and incubation in 4°C for 30 min shaking after the sample was transferred in a new 1,5 ml eppendorff tube and 560 ml of water/methanol mixture (3:1 v:v) was added. This led to the formation of two phases: a lipophilic phase and a polar phase. After the addition of methanol-water mixture, the sample was vortexed for 10 min and centrifuged for 10 min at 4°C at the speed 12700 rpm. The upper lipophilic phase was collected and vacuum dried (1,5 h, 30°C Concentrator plus, Eppendorf) and stored at - 80°C before measurement. This protocol is based on the protocol described by Giavalisco et al. 2011. For FA’s quantification 5 isotopically labeled lipid internal standards were added to the extraction mixture (3 ug of each per sample): Oleic acid-^13^C_18 (#_490431 Sigma Aldrich_),_ Palmitic acid-^13^C_16 (#_605573 Sigma Aldrich, Stearic acid-^13^C_18 (#_605581 Sigma Aldrich, **18:1-d7**

LPC _#_791643 Avanti, and 15:0-18:1-d7 DG _#_791647 Avanti).

For fatty acid extraction, lipid extracts were resuspended using 200 **u**l of a mixture of methanol and 6% KOH (4 : 1, v : v). The tubes were incubated for 2 h at 60°C with continuous shaking (1800 rpm). After cooling to room temperature, 100 **u**l of saturated NaCl solution was added. The reaction mixture was acidified by the addition of 50 **u**l of 29% HCl. Tubes were vortexed thoroughly and spun for 30 s at full speed using a table centrifuge. The FAs were extracted with 200 **u**l of chloroform**–**heptane mixture (1 : 4, v : v). After vortexing and 15 s of centrifugation, the organic phase was collected. The extraction with the chloroform/heptane mixture was repeated the second time and the collected FA-containing organic phases were combined. The extract was washed by the addition of 200 **u**l of water followed by short vortexing on a table vortex and 15-sec centrifugation at 12700 rpm, which resulted in two phases. Finally, the organic upper phase was collected, dried in vacuum conditions (30min, 30°C Concentrator plus, Eppendorf) and stored at - 80°C before the measurements.

**UPLC-MS profiling**

For the FA profiling, spectrum acquisition in ESI- positive mode was performed. For UPLC separation gradient mobile phases consisted of two solvents.

For all FAs profiling excluding 18:1 and 18:2: Solvent A: 1%1 M NH4Ac and 0.1% formic acid in water; and solvent B, acetonitrile/isopropanol (7 : 3, 1% 1 M NH4Ac, 0.1% formic acid), with an injection volume of 3 ul. The following gradient profile was applied: 1 min, 55% B; 3 min, linear gradient from 55% B to 80% B; 8 min, linear gradient from 80% B to 85% B; 3 min, linear gradient from 85% B to 100% B. After washing the column for 4 min 50 sec with 100% B, the mixture was set back to 55% B and the column was re-equilibrated for 4 min 10 sec (24.5 min total run time), with a flow rate of the mobile phase of 400 ll min. Final sample dilution: 1:5.

For the 18:1 and 18:2: Solvent A, 1%1 M NH4Ac and 0.1% acetic acid in water; and solvent B, acetonitrile/isopropanol (7 : 3, 1% 1 M NH4Ac, 0.1% acetic acid), with an injection volume of 3 ul. The following gradient profile was applied: 50 sec, 55% B; 1 min, linear gradient from 55% B to 75% B; 5 min, linear gradient from 75% B to 89% B; 1 min 10 sec, linear gradient from 89% B to 100% B. After washing the column for 2 min with 100% B, the mixture was set back to 55% B and the column was re-equilibrated for 1 min 50 sec (11.5 min total run time), with a flow rate of the mobile phase of 400 ll min. Final sample dilution 1:400.

For all other lipid profiling spectrum acquisition in ESI+ positive mode was performed. For the UPLC separation the following solvent system was used: solvent A: 1%1 M NH4Ac and 0.1% formic acid in water; and solvent B, acetonitrile/isopropanol (7 : 3, 1% 1 M NH4Ac, 0.1% formic acid), with an injection volume of 3 ul. With the same gradient parameters that were used for FAs profiling. Final sample dilution: 1:25.

For the 6 lines which were collected from 3 different years ESI - profiling with 1:10- dilution and ESI+ profiling in 1:25 and 1:3 dilutions were performed.
