# Supplementary material for: Genotyping and lipid profiling of 601 cultivated sunflower lines reveals novel genetic determinants of oil fatty acid content
Source: BMC Genomics. 2021 Jul 5;22:505. doi: 10.1186/s12864-021-07768-y (PMC8256595; doi:10.1186/s12864-021-07768-y)
Supplement: Supplementary file 23 — Additional file 23: AppendixS1. Description of the lines used in the study. [file 12864_2021_7768_MOESM23_ESM.docx]

Appendix S1

Inbred lines from VIR collection were obtained by repeated self-pollination of the interspecific and industrial hybrids obtained from different countries (8-25 generations of inbreeding). The majority of the lines made based on first heterosis hybrids Soldor (VIR704), Sunbred 265 from France (VIR630, 631) F1 (SW536xW635(France)) (VIR 636, 655,700, 734). Two lines–fertility restorer lines VIR 453 and VIR 658, obtained by continues self-pollination of wild *H. annuus.* In the study we used lines with various morphological features: different height, branching, leaf shape, leaf and flowers color, vegetation period and resistance to downy mildew.

VNIIMK lines by origin close to VIR lines but they are different in terms of morphological traits combination, because an inbreeding was performed by different researchers in each generation. VNIIMK lines are contrast by fatty acid composition.

Agroplasma company collection presented mostly by fertility restorer lines resistant to the broomrape (*Orobanche* *cumana*) and a smaller fraction of sterility maintaining lines. Lines were obtained by self-pollination of modern industrial hybrids.
